# Physiological and Proteomic Analyses of Two Acanthus Species to Tidal Flooding Stress

**DOI:** 10.3390/ijms22031055

**Published:** 2021-01-21

**Authors:** Yi-ling Liu, Hai-lei Zheng

**Affiliations:** Key Laboratory of the Ministry of Education for Coastal and Wetland Ecosystems, College of the Environment and Ecology, Xiamen University, Xiamen 361005, Fujian, China; liuyiling@stu.xmu.edu.cn

**Keywords:** Acanthus species, flooding stress, physiological, comparative proteomics analyses, carbon, energy metabolism

## Abstract

The mangrove plant *Acanthus ilicifolius* and its relative, *Acanthus mollis*, have been previously proved to possess diverse pharmacological effects. Therefore, evaluating the differentially expressed proteins of these species under tidal flooding stress is essential to fully exploit and benefit from their medicinal values. The roots of *A. ilicifolius* and *A. mollis* were exposed to 6 h of flooding stress per day for 10 days. The dry weight, hydrogen peroxide (H_2_O_2_) content, anatomical characteristics, carbon and energy levels, and two-dimensional electrophoresis coupled with MALDI-TOF/TOF MS technology were used to reveal the divergent flooding resistant strategies. *A. ilicifolius* performed better under tidal flooding stress, which was reflected in the integrity of the morphological structure, more efficient use of carbon and energy, and a higher percentage of up-regulated proteins associated with carbon and energy metabolism. *A. mollis* could not survive in flooding conditions for a long time, as revealed by disrupting cell structures of the roots, less efficient use of carbon and energy, and a higher percentage of down-regulated proteins associated with carbon and energy metabolism. Energy provision and flux balance played a role in the flooding tolerance of *A. ilicifolius* and *A. mollis*.

## 1. Introduction

The physical characteristics of soil influence a variety of physiological and biochemical processes of plants. The leaves and roots of terrestrial plants absorb molecular oxygen from air and land, respectively [1]. Previous studies have shown that flooding stress is a widespread phenomenon that inhibits plant growth and production [2]. Continuous and heavy rainfall causes soil pores saturated with excess water, inducing oxygen deficiency in plant roots [1,2]. Meanwhile, the roots that are subjected to flooding stress may inhibit photosynthesis, including a decrease of photosynthetic electron transport chain and an increase in the level of reactive oxygen species (ROS) [3]. A shift of aerobic respiration to anaerobic respiration reduced the availability of the adenosine triphosphate (ATP) in plants [4] and increased the content of ethanol [5].

Some plants have evolved morphological, physiological, and metabolic adaptation strategies to ensure survival under flooding stress [6,7]. For example, maize develops an extensive aerenchyma system to facilitate gas transport apart from adventitious roots [6]. Rice retains a gas-associated film to facilitate oxygen uptake to survive under flooding [7]. Mangrove plants, such as *Kandelia obovata*, *Sonneratia apetala*, *Aegiceras corniculatum*, and *Rhizophora stylosa* develop specialized roots for gaseous exchange [8]. Soybean (PELBR10-6000) increased the level of CO_2_ assimilation rate and readily responded to the lack of energy by activating the fermentative enzymes and alanine aminotransferase, when it was allowed to recover for additional seven days after flooding treatment [9], indicating that the capacity to quickly resume the normal energy level is crucial in tolerating flooding stress [10].

A comparative study of species is one of the important methods to determine the mechanism of stress-resistance. The difference in fermentative enzymes and alanine aminotransferase activity resulted in different responses to energy deficiency between two soybean genotypes under flooding conditions [9]. A comparison of *Alternanthera philoxeroides* with *Hemarthria altissima* showed that plants could adapt to wetland habitats, in which water levels fluctuate, by maintaining the functionality of the photosynthetic apparatus [11]. Waterlogged *Phalaris aquatica* and *Festuca arundinacea* regained growth during the recovery period compared with *Dactylis glomerata* and *Bromus catharticus* [12]. In addition, the favorable alleles of related species are more comfortable to introduce to improve crops [13]. The transfer of resistance genes between *Sinapis alba* and *Brassica* species by somatic and sexual hybridization has been accomplished [14].

The mangrove plant, *Acanthus ilicifolius*, has remarkable morphology and physiology [15]. *A. ilicifolius* is mainly distributed in Australia, Australasia, and the southeastern Asia intertidal zone and has numerous medicinal properties [16]. Previous findings showed that untreated or submerged *A. ilicifolius* over 3 h per day was not conducive to the growth [17]. Meanwhile, the effect of flooding stress on *A. ilicifolius* at the molecular level is not well elucidated. Acanthus belongs to the Acanthaceae family and is the only genus that comprises of both terrestrial and aquatic species [15]. As the Acanthus model plant, *A*. *mollis* is native to the Mediterranean region from central Europe and northwest Africa [18]. *A. mollis* was recently introduced into China and used as a medicinal plant in traditional medicine [18,19]. The extracts of *A. mollis* tissues have been used for the treatment of inflammation and cancer problems [20]. However, the flooding tolerance of *A. mollis* has not yet been described. Since non-model plants lack a genetic transformation system to elucidate the metabolic mechanism, proteome and transcriptome are useful to provide powerful information about the metabolic pathways of non-model plants. The protein, as the functional executor, is closely related to physiological changes. In our previous study, we have reported the flooding tolerance of the leaves of *Avicennia marina* using comparative proteomic analyses [21]. Hence, evaluating the differentially expressed proteins (DEPs) of *A. ilicifolius* and *A. mollis* under tidal flooding stress is essential to fully exploit and benefit from their medicinal values.

Paraffin sections, physiological index measurements, and two-dimensional electrophoresis (2-DE) technique were performed on the leaves and roots of *A. ilicifolius* and *A. mollis* under tidal flooding stress. Our results first provide the anatomical characteristics, carbon and energy levels, and proteomic information about *A. ilicifolius* and its relative, *A. mollis*, under tidal flooding stress.

## 2. Results

### 2.1. Relative Dry Weight and H_2_O_2_ Content of Acanthus Species under Tidal Flooding Stress

**Figure 1:**
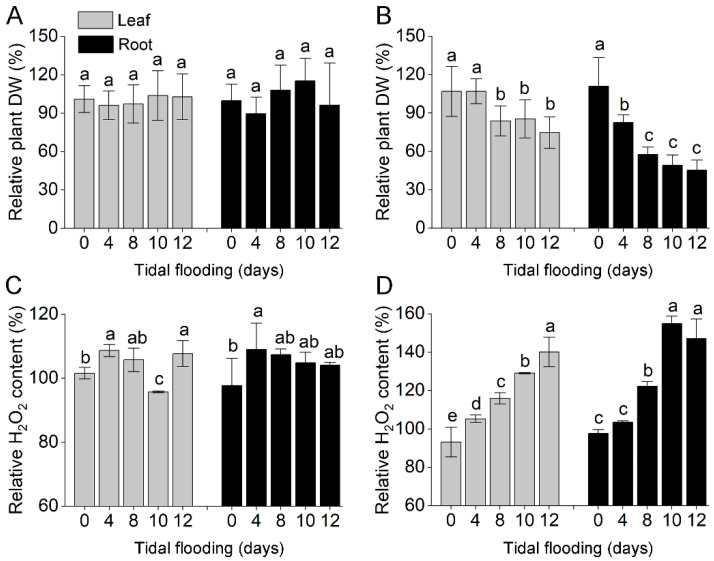
(**A**) Relative plant dry weight of *A. ilicifolius*. (**B**) Relative plant dry weight of *A. mollis*. (**C**) Relative H_2_O_2_ content of *A. ilicifolius*. (**D**) Relative H_2_O_2_ content of *A. mollis*. Gray represents leaves; black represents roots. Data are shown as means ± SD from three independent biological replicates. Means marked with the same letter were not different from each other, but were different from means marked with a different letter; *p* < 0.05. The different bars indicate the different tissue of plant.

The two species changed the relative dry weight and H_2_O_2_ content to differing extents under tidal flooding stress. The relative dry weight of *A. ilicifolius* had no change in both the leaf and root tissues (Figure 1A). The relative H_2_O_2_ content was significantly decreased on the tenth day in *A. ilicifolius* leaves and showed no significant differences from day four to 12 in *A. ilicifolius* roots (Figure 1C). Tidal flooding treatment significantly decreased the relative dry weight (Figure 1B) and increased the relative H_2_O_2_ content of *A. mollis* in both tissues (Figure 1D). Overall, the relative dry weight (expressed as a percentage of the control) of *A. ilicifolius* was significantly higher compared to that of *A. mollis* from day eight to 12 (Supplementary Table S1).

### 2.2. Effect of Tidal Flooding on the Phenotype and Anatomical Characteristics of Acanthus Species

**Figure 2:**
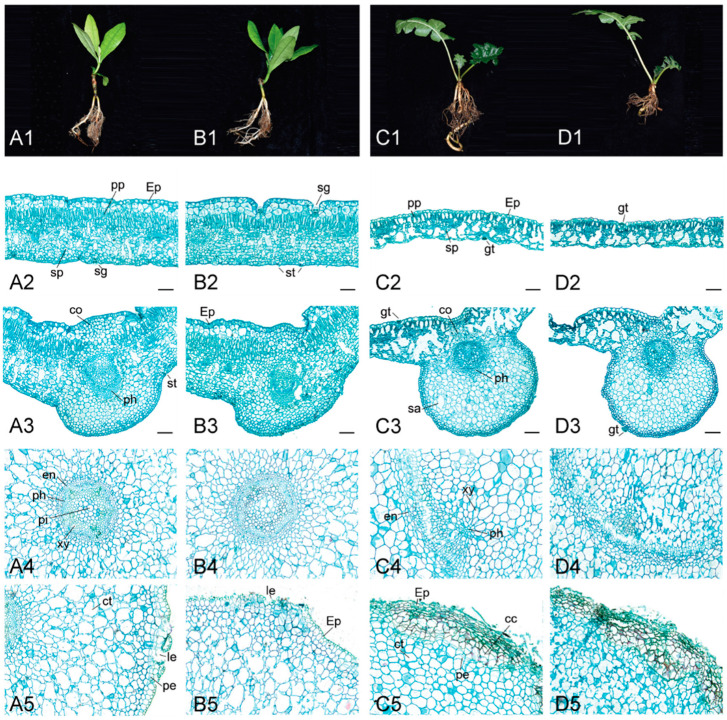
Phenotypic and anatomical changes of *A. ilicifolius* and *A. mollis* exposed to tidal flooding for 10 days. (**A1**–**A5**) *A. ilicifolius* plants on control treatment, (**B1**–**B5**) *A. ilicifolius* plants under tidal flooding stress, (**C1**–**C5**) *A. mollis* plants on control treatment, (**D1**–**D5**) *A. mollis* plants under tidal flooding stress. Row 1: The phenotypic of Acanthus species, Row 2: The cross-section of the leaf blade, Row 3: The main vein of the leaf, Row 4: Stele of root, Row 5: Epidermis of root. Root sections, about 5 cm from root tip, photos of optical microscopes. Cross-sections with thickness of 10 mm were made and stained with safranine and fast green. cc: Cork cambium; co: Collenchyma; ct: Cortex; en: Endodermis; Ep: Epidermis; gt: Glandular trichome; le: Lenticel; pe: Periderm; ph: Phloem; pi: Pith; pp: Palisade parenchyma; sa: Schizogenous aerenchyma; st: Stomata; sp: Lacunar parenchyma; xy: Xylem. Bars = 100 μm.

The phenotype and anatomical characteristics of the two species are shown in Figure 2. After 10 days of tidal flooding treatment, there was no significant change in *A. ilicifolius* (Figure 2(A1,B1)), while the length of *A. mollis* roots became shorter (Figure 2(C1,D1)).

The leaf blade of *A. ilicifolius* consisted of the upper epidermis, upper multiple epidermises, palisade parenchyma, spongy parenchyma, lower epidermis, and salt gland (Figure 2(A2)), whereas that of *A. mollis* showed a different structure. The upper and lower epidermises of *A. mollis* were all single-layered and had glandular trichome (Figure 2(C2)). *A. ilicifolius* roots consisted of the endodermis, epidermis, xylem, phloem, pith, cortex, lenticel, and periderm (Figure 2(A4,A5)), whereas the roots of *A. mollis* contained cork cambium (Figure 2(D5)).

In the leaf blade of *A. ilicifolius*, the vein phloem possessed a hollow cavity that was enlarged after tidal flooding treatment (Figure 2(B3)). The control group of *A. mollis* exhibited schizogenous aerenchyma in the leaf vein, which disappeared after tidal flooding treatment. The periderm is a secondary protective tissue that protects plant roots from bacterial infections [22]. In *A. mollis* roots, the pith parenchyma cells were damaged and the periderm cells were ruptured under tidal flooding stress (Figure 2(D4,D5)).

### 2.3. Identification and Quantification of Tidal Flooding-Responsive Proteins

**Figure 3:**
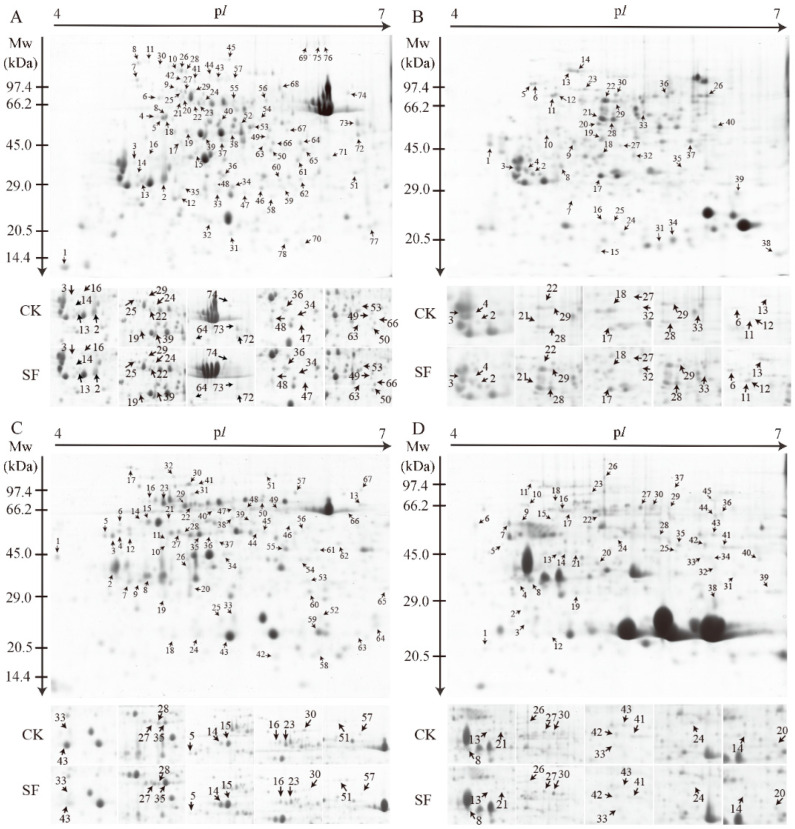
Two-dimensional (2-DE) analysis of proteins extracted from (**A**) *A. ilicifolius* leaves, (**B**) *A. ilicifolius* roots, (**C**) *A. mollis* leaves, and (**D**) *A. ilicifolius* roots. The numbers correspond with the spot ID, mentioned in Supplementary Tables S2–S5. The isoelectric point (p*I*) and molecular weight (MW) in kilodaltons are indicated on the top and left of the gel, respectively. CK and SF represent the control group and soil tidal flooding stress, respectively.

Representative 2-DE gels of the leaves and roots of the two species are shown in Figure 3. In *A. ilicifolius*, approximately 78 and 40 spots were identified from leaves and roots, respectively (Figure 3A,B; Supplemental Tables S2, S3 and S6). In *A. mollis*, about 67 and 45 spots were identified from leaves and roots, respectively (Figure 3C,D; Supplemental Tables S4–S6).

**Figure 4:**
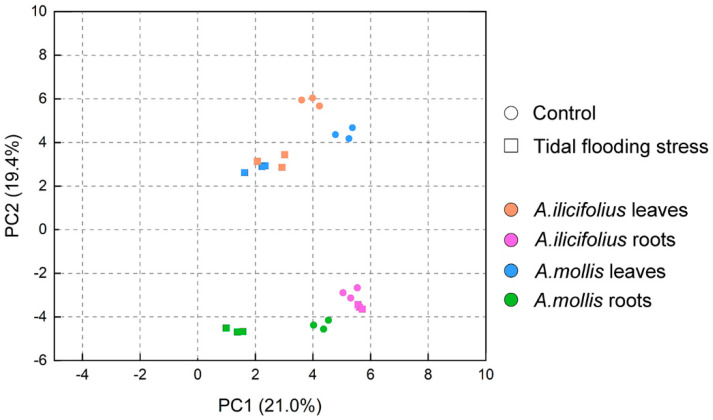
Principal component analysis (PCA) of total proteome data for the tissues of *A. ilicifolius* and *A. mollis*. Percentage variance for each principal component is given.

To understand the global relationship between samples, PCA was performed to evaluate the similarity between the samples (Figure 4) [23]. In *A. ilicifolius* leaves, the control group and the tidal flooding group were well separated from each other in the dimension of the second component. In *A. mollis* tissues, the control group and the tidal flooding group were well separated from each other in the dimension of the first component. Leaves and roots were well separated from each other in the dimension of the second component. However, the two species were not well separated from each other. PC1 explained 21.0% and PC2 19.4% of total variance.

### 2.4. Functional Classification of DEPs

**Figure 5:**
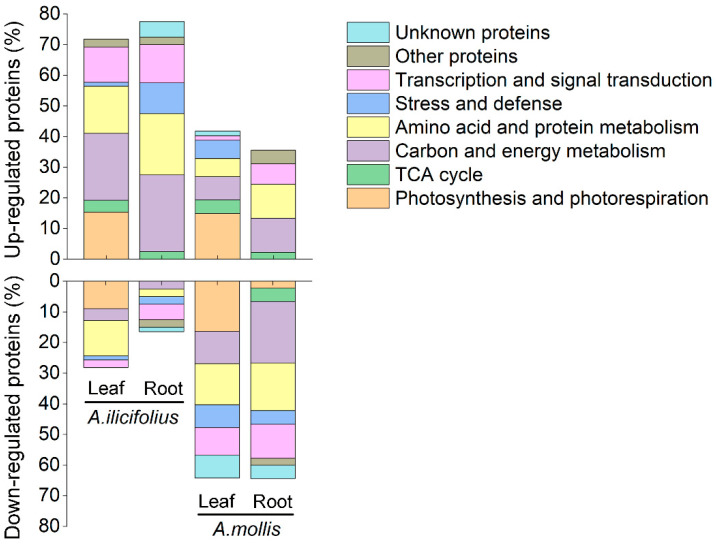
Functional classification analysis of the differentially expressed proteins (DEPs) of *A. ilicifolius* and *A. mollis*. The detailed information for each spot is shown in Supplementary Tables S2–S5.

More proteins were up-regulated in *A. ilicifolius* than in *A. mollis* tissues (Figure 5, Supplemental Table S7). A higher percentage of up-regulated proteins were found in carbon and energy metabolism and amino acid and protein metabolism in *A. mollis* leaves, while in transcription and signal transduction in *A. ilicifolius* leaves. In addition, *A. ilicifolius* leaves had a lower percentage of up-regulated proteins associated with stress and defense. Overall, *A. ilicifolius* tissues had a high percentage of up-regulated proteins and a low percentage of down-regulated proteins associated with carbon and energy metabolism. Meanwhile, a higher percentage of down-regulated proteins of *A. mollis* leaves were associated with carbon and energy metabolism, stress and defense, and transcription and signal transduction (Figure 5, Supplemental Table S7).

Compared with *A. mollis* roots, a higher percentage of up-regulated proteins of *A. ilicifolius* roots were associated with carbon and energy metabolism, amino acid and protein metabolism, stress and defense, and transcription and signal transduction. In *A. mollis* roots, there was a higher percentage of down-regulated proteins in all pathways (Figure 5, Supplemental Table S7).

### 2.5. Identification of Hub Proteins in Acanthus Species

**Figure 6:**
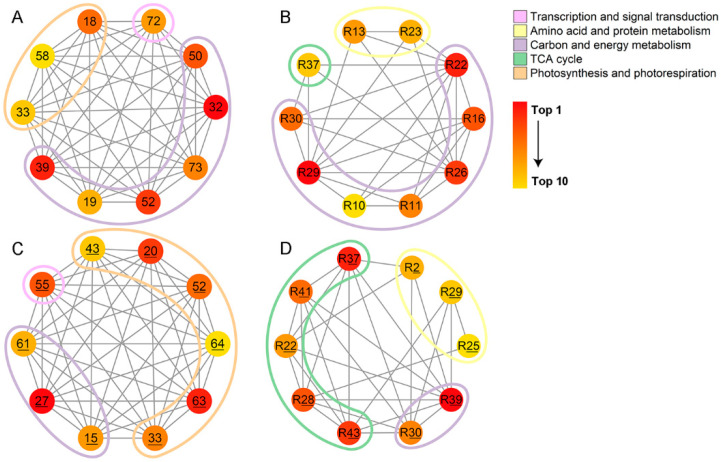
Top 10 hub proteins in network of (**A**) *A. ilicifolius* leaves, (**B**) *A. ilicifolius* roots, (**C**) *A. mollis* leaves, and (**D**) *A. mollis* roots ranked by Matthews correlation coefficient (MCC) method. R represents the root tissue. Underlined numbers represent *A. mollis* tissues.

Because of the lack of genome information on *A. ilicifolius* and *A. mollis*, the DEPs of the two species were annotated based on the existing non-redundant protein sequence database (NR). Based on our previous studies [21] and homologous protein distribution analysis (Supplementary Figure S3), *Arabidopsis thaliana* was used to assemble the protein-protein interaction (PPI) network of *A. ilicifolius* and *A. mollis*. The top-ten hub proteins were identified with a degree score of CytoHubba and displayed in Figure 6. The hub proteins of *A. ilicifolius* tissues were mostly associated with carbon and energy metabolism (Figure 6A,B), whereas those of the *A. mollis* tissues were mostly associated with photosynthesis and photorespiration and the TCA cycle (Figure 6C,D).

### 2.6. Tidal Flooding Stress Influences the Energy Status Level of A. ilicifolius and A. mollis

**Figure 7:**
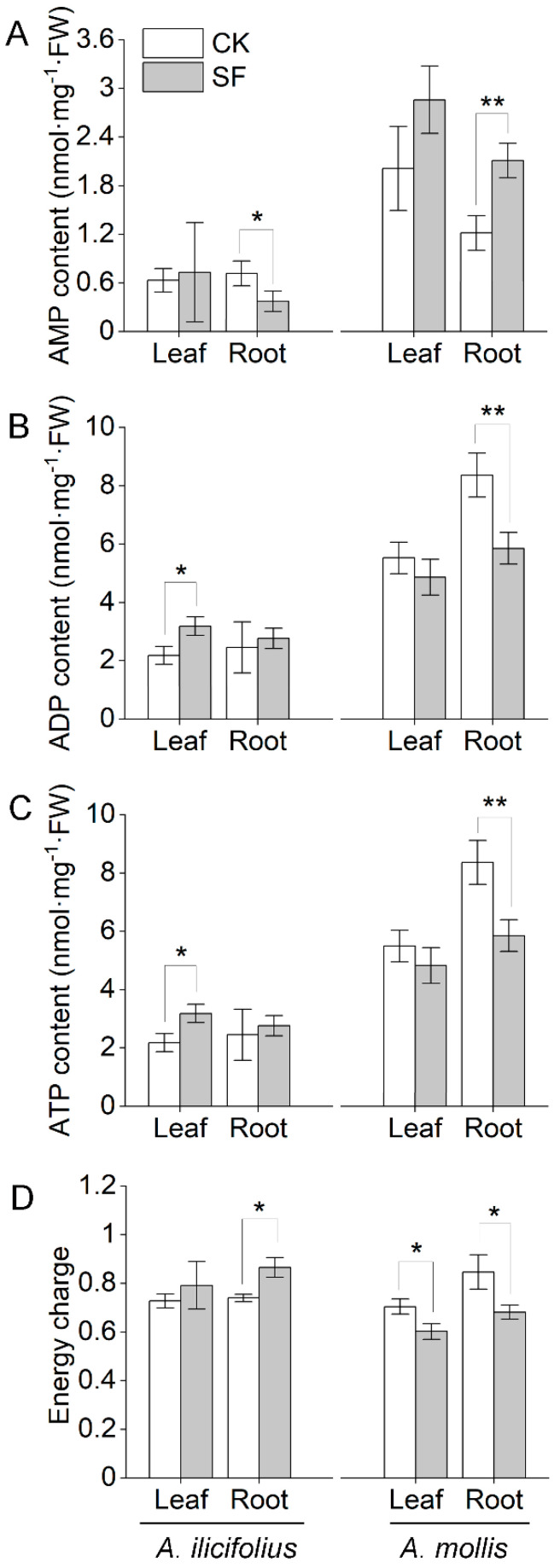
Effects of tidal flooding stress on (**A**) adenosine monophosphate content, (**B**) adenosine diphosphate content, (**C**) adenosine triphosphate content, and (**D**) energy charge of *A. ilicifolius* and *A. mollis*. * and ** indicate significant difference at the 0.05 level and the 0.01 level, respectively. CK and SF represent the control group and soil tidal flooding stress, respectively.

The further comparison demonstrated that *A. mollis* had a higher adenosine monophosphate (AMP) content, adenosine diphosphate (ADP) content, and ATP content than *A. ilicifolius* in the control group (Supplementary Figure S4). *A. ilicifolius* promptly responded to flooding stress by significantly increasing ADP and ATP contents in the leaves (Figure 7B,C). However, AMP and ATP contents were significantly decreased in *A. mollis* roots (Figure 7B,C) under tidal flooding stress. The energy charge represents the energy status of biological cells [24]. Whereas the energy charge of *A. ilicifolius* roots was significantly increased under tidal flooding stress, it was significantly decreased in *A. mollis* tissues (Figure 7D).

### 2.7. Tidal Flooding Stress Influences the Total Soluble Sugar and Starch Contents of Acanthus Species

**Figure 8:**
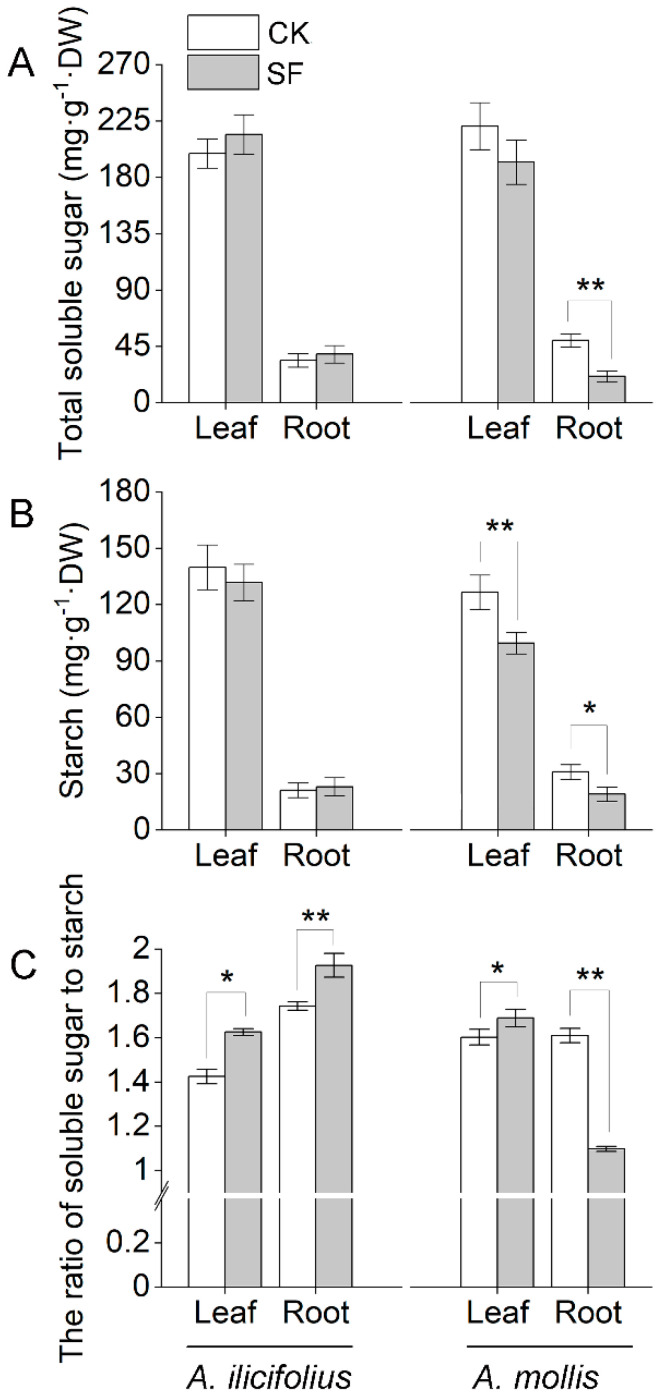
The concentration of (**A**) soluble sugar, (**B**) starch and (**C**) the ratio of soluble sugar to starch for *A. ilicifolius* and *A. mollis* under tenth day tidal flooding stress. * and ** indicate significant difference at the 0.05 level and the 0.01 level, respectively. CK and SF represent control and soil tidal flooding stress, respectively.

There was no significant change in the content of total soluble sugar and starch of *A. ilicifolius* tissues under tidal flooding stress (Figure 8A,B). Nevertheless, there was significant tidal flooding tolerance in the ratio of soluble sugar to starch in *A. ilicifolius* tissues (Figure 8C). The total soluble sugar content, starch content, and the ratio of soluble sugar to starch were lower in *A. mollis* tissues than in the control group, except for the ratio of soluble sugar to starch in the leaves (Figure 8A–C).

## 3. Discussion

### 3.1. Differences in Tissue Tolerance between Acanthus Species

Unlike previous findings in the mangrove, *A. marina*, seedlings [25], the upper and lower epidermises of *A. ilicifolius* showed no change with prolonged waterlogging duration in the present study (Figure 2(B2,B3)). The leaf anatomical features of *A. mollis* were also relatively less susceptible to tidal flooding stress within a short span. The leaf anatomy plays an important role in determining photosynthetic capacity. Herbaceous plants with high photosynthetic capacity usually have thinner epidermis, leading to high values of mesophyll conductance [26]. The mangrove leaf exhibited a range of xeromorphic features, including thick epidermis and wax coatings [25]. Therefore, like other mangrove plants, *A. ilicifolius* leaves are likely to regulate the Calvin cycle to resist the tidal flooding stress (Supplementary Table S2). A comparative analysis showed that palisade and spongy tissue that were loosely arranged with large spaces and epidermis were thinner in *A. mollis* leaf, making CO_2_ entry easier.

*A. ilicifolius*, mainly distributed in the foreshore seaward region, was found to develop a high percentage of schizogenous aerenchyma to facilitate efficient internal oxygen transfer [27]. According to previous study, the mangrove species appeared to higher waterlogging tolerance when the aerenchyma formation was induced [28]. The aortic root anatomy of *A. ilicifolius* was not affected by the tidal flooding stress. The special anatomical structure of roots was not the main reason for *A. ilicifolius* to tolerance tidal flooding stress at the early stage. Water and minerals transport from the root system to the aerial portions via the xylem tissue. The phloem translocates photosynthetic products from mature leaves to roots and redistributes water and various compounds throughout the plant body [29]. In *A. mollis*, the aortic root anatomy exhibited broken xylem, phloem, and periderm tissues, indicating a negative influence on the allocation and partitioning of photosynthetic products.

### 3.2. Effect of Tidal Flooding on the Photosynthesis of Acanthus Species

The proportion of photosynthesis-related proteins within the total DEPs of *A. ilicifolius* leaves was close to that of *A. mollis* leaves (Figure 5). The abundance of oxygen-evolving enhancer protein (OEE, spot 18) showed an increasing trend in *A. ilicifolius* leaves under tidal flooding stress. Oxygen-evolving complex (OEC) proteins are degraded and release OEE as a degradation product to promote the plant to adapt to the adverse conditions [30]. OEE is a subunit of the OEC of photosystem II in the chloroplast [31] considered to be directly involved in photosynthesis. It is suggested that decreased OEE abundance (spot 20, 33) might negatively affect *A. mollis* leaves. Most of chlorophyll a/b-binding proteins increased in *A. ilicifolius* leaves (spot 12, 13, 58) but decreased in *A. mollis* leaves (spot 43) under tidal flooding stress. However, electron transport chain proteins, such as ferredoxin-nicotinamide adenine dinucleotide phosphate (NADP) reductase (spot 52), cytochrome b6-f complex iron-sulfur subunit 1 (spot 63), and photosystem I reaction center subunit IV b (spot 64), increased in *A. mollis* leaves, promoting photosynthetic electron transport under tidal flooding stress [31].

One fraction of the captured light energy is used to reduce NADP^+^ to reduced nicotinamide adenine dinucleotide phosphate (NADPH) and the other fraction is used for light-dependent ATP synthesis. The proteomic data showed that chloroplast ATP synthesis was decreased in *A. mollis* leaves (spot 23, 29, 30, 31). ATP-dependent zinc metalloprotease FTSH2 (FtsH2, spot 41, 42), which is involved in the turnover of the ΦPSII reaction center D1 protein [32], was increased in maize to protect chloroplast photosynthesis under heat stress [33]. Herein, increased FtsH2 abundance had a positive effect on tidal flooding tolerance of *A. ilicifolius* leaves.

### 3.3. Effect of Tidal Flooding on Carbon and Energy Metabolism of Acanthus Species

Plants change their energy metabolism pathways to meet the increased demands for survival [34]. CO_2_ fixation is performed through the Calvin cycle to drive sugar production and energy storage in plants [33]. The activation state of RuBisCO, a key enzyme in the Calvin cycle, is regulated by RuBisCO activase [35]. RuBisCO activase increased in both *A. ilicifolius* (spot 5 and 19) and *A. mollis* (spot 14, 15, 21, 28, 35, and 36). The abundance of RuBisCO large subunits increased in *A. ilicifolius* (spots 7, 9, and 15) but decreased in *A. mollis* (spots 3, 5, 22, 25, and 40). A similar result was observed in *Trifolium* species, the waterlogging-sensitive species exhibited a higher reduction of RuBisCO large subunits expression [34].

A higher soluble sugar concentration (Figure 8A) in flooded *A. ilicifolius* plant is not solely due to photosynthesis but also the conversion of carbohydrates from starch to sugar (Figure 8C). Fructokinase (spot R32) regulates starch synthesis coordinately with sucrose synthase and plays a key role in starch accumulation in tomato fruit [36], which exhibited a three-fold increase in *A. ilicifolius* roots under tidal flooding treatment. Decreased alpha-galactosidase (spot R7), beta-galactosidase (spot R26), beta-glucosidase (spot R10), and UDP-glucose 4-epimerase family protein (spot R35) abundance had adverse effects on *A. mollis* roots under tidal flooding stress, indicating the inhibition of polysaccharide catabolism and the interconversion of hexoses (glucose/galactose) in *A. mollis* roots under tidal flooding stress [37,38,39].

Glycolysis and the TCA cycle mainly provide energy for plant growth and development [40]. Pyruvate produced via the glycolytic pathway into the TCA cycle and the consequent electrons is transferred along an electron transport chain and then return to the mitochondrial matrix via ATP synthase [41]. Similar to the proteomic data of a previous study [21], increased glycolysis and TCA cycle-related protein abundances contributed to the defense system of *A. ilicifolius* tissues tidal flooding stress. Meanwhile, increased V-ATPase abundance was helpful to maintain the cytosolic pH homeostasis and provide free energy to establish a proton motive force across membranes in *A. ilicifolius* tissues [42]. Mitochondrial ATP synthase was decreased in *A. ilicifolius* leaves (spot 24, 32) but increased in *A. ilicifolius* roots (spot R16, R22, R26, R29), indicating that the requirement of ATP decreased accordingly. This implied that high levels of ATP synthase abundance might not be needed in *A. ilicifolius* leaves. In *A. mollis*, the abundance of glycolysis and TCA cycle-related proteins mostly showed an increase in leaves and a decrease in the roots under tidal flooding stress. Due to the low efficiency of energy conservation under fermentation, an increased rate of glycolysis is required to sustain ATP production necessary for cell survival [43]. The abundance of malate dehydrogenase (ADH; spot R36, R44, R45) increased with a concomitant reduction in glycolysis and TCA cycle-related proteins (spot R28, R37), indicating the low efficiency of energy conservation in *A. mollis* roots under tidal flooding stress [44]. Physiological data also showed the ATP level and energy charge of *A. mollis* roots became too low to sustain the basal metabolic requirement of roots (Figure 7C,D).

### 3.4. Effect of Tidal Flooding on Nutrient Assimilation and Protein Metabolism of Acanthus Species

Sugar metabolism provides sufficient energy to amino acid metabolism and intermediates from glycolysis can be utilized as precursors for the synthesis of amino acids [45]. Increased abundances of glutamine synthetase (spot 53), glutamate-ammonia ligase family protein (spot 66, R34, R40), glycine dehydrogenase (spot 75, 76), MTA/SAH nucleosidase (spot 37), and S-adenosyl methionine synthetase (spot R33) were considered to contribute to the production of amino acids in the cell of *A. ilicifolius* tissues [45,46,47]. In *A. mollis* tissues, nitrogen metabolism-related enzymes, such as glutamine synthetase (spot 10, 44) and arginase (spot 56), showed an increase. However, decreased sulfate adenylyl transferase (spot 48), cysteine synthase (spot 34) and adenosyl homocysteinase (spot 50) abundances indicated adverse effects on producing cysteine from sulfate in *A. mollis* leaves [12,48].

Plant tissue exposure to abiotic stress induces protein damage in cells. Increased proteasome subunit alpha type (spot 17) and proteasome subunit beta type (spot 59) abundances were observed in *A. ilicifolius* to break down damaged proteins under tidal flooding stress [49]. Furthermore, heat shock proteins (spots 10, 11, 26, 28, 29, and R13), RNA-binding protein NOB1 (spot 47), protein disulfide isomerase (spot 27), which participate in plant fitness, the biogenesis of 40S ribosomal subunits and the formation of disulfide bonds during protein folding [50,51], mostly increased in *A. ilicifolius* tissues under tidal flooding stress. In *A. mollis*, the abundance of proteasome subunit alpha type (spot 11, R14) and polyubiquitin (spot R13) were decreased, but 26S protease regulatory subunit 6A homolog (spot R15) and 20S proteasome alpha subunit (spot R25) increased during the flooding stage. In addition, protein synthesis essentially requires ribosomes, which play a distinct role in all living cells [52]. The tidal flooding stress decreased 60S ribosomal-related proteins (spot R1, R38) abundance, suggesting inhibition of protein synthesis in *A. mollis* roots.

14-3-3 protein, which takes part in the regulation of carbon and nitrogen metabolism [53], showed an increase in *A. ilicifolius* (spot 3, 14, R2, and R3) but a decrease in *A. mollis* (spot 9, R4, and R8), indicating tidal flooding stress damage of the signal pathway of carbon and nitrogen metabolism in *A. mollis* tissues.

### 3.5. Effect of Tidal Flooding on Antioxidative Defense System of Acanthus Species

The antioxidative defense system has a stronger reactive oxygen scavenging capacity to mitigate oxidative damage under flooding stress [54]. Our data suggested that most of the antioxidant enzyme-related proteins, including l-ascorbate peroxidase (spot 36, R17), thioredoxin (spot R15, R31), monodehydroascorbate reductase (spot R20), and catalase (spot R36), were increased in *A. ilicifolius* under tidal flooding stress. The ascorbate-glutathione cycle together with 2-Cys peroxiredoxin are relevant systems in detoxifying reactive oxygen in stressed plants [55]. It has been reported that most of the enzymes (spot 2, R7, 24, and 49) involved in this process decrease in *A. mollis*.

In addition, annexin genes have been reported to have peroxidase activity [56]; it has been hypothesized that elevated abundance of annexin D5-like (spot 42) together with glutathione S-transferase (spot 60) modulate endogenous ROS levels in *A. mollis* leaves under tidal flooding stress. Moreover, glutathione S-transferase in protein regulation via S-glutathionylation, as a post-translational modification, have been reported in plants [57].

## 4. Materials and Methods

### 4.1. Plant Material and Experimental Setup

Experiment materials were obtained from vegetative propagation. The stems of *A. ilicifolius* (9–12 mm in diameter and 10–20 cm in length) were collected from the Zini mangrove forest (117°91′ E, 24°45′ N), south of the Jiulong River Estuary, Fujian Province, China. The roots of *A. mollis* were collected from a mother plant that was planted in the greenhouse for one year. The explants of *A. ilicifolius* and *A. mollis* were placed in pots (19 cm in diameter and 20 cm in depth) with soil plus vermiculite in a ratio of 3:1. The growth of cuttings is shown in Supplementary Figure S1. Hoagland nutrient solution of 1/8 strength with rooting hormone powder was used to promote the growth of stems and adventitious roots. In the first two months, the cuttings were grown under controlled conditions: Temperature (28 ± 2 °C), weak light, and relative humidity (60 ± 5%). The pots were then transferred to a new condition with 1000 μmol·m^−2^·s^−1^ light intensity, 12 h light period day^−1^, and 28 ± 2 °C temperature.

After growth for five months, uniform and healthy plants were selected for further analysis. The plants were randomly divided into two groups. The soil water content of the control group was kept at 65 ± 5% and regulated by the oven drying method. The pots were placed inside 50 L plastic containers maintaining a 1–2 cm water layer above the soil surface (Supplementary Figure S2) and treated with flooding stress for 6 h per day. All the pots perforated at the bottom to ensure proper drainage. The dry weight of the leaves and roots samples were taken every two days. The extraction of protein and RNA, paraffin sectioning, and the measurement of energy AMP, ADP, ATP, and hydrogen peroxide (H_2_O_2_) content were performed on the tenth day.

### 4.2. Determination of the Dry Weight

Five plants were randomly selected, washed with distilled water, and divided into two parts (leaves and roots). The oven-dried (at 70 °C for 72 h) leaves and roots samples were measured for dry weight (DW) in grams (g) using an electric weight balance. Relative plant DW = Wt/Wo × 100%, where Wt is the dry weight under tidal flooding treatment and Wo is the dry weight under control conditions [58]. Each measurement was repeated three times with five replications per treatment.

### 4.3. Anatomical Features of Leaves and Roots

The leaf center and mature root were collected from *A. ilicifolius* and *A. mollis* for paraffin sectioning. The fresh tissues were fixed in formalin-acetic acid-alcohol (FAA) solution for 72 h, followed by 6 h in an ethanol series (50–100%) to being embedded in paraffin, sectioned, and stained with 1% aqueous safranin and 0.5% fast green. Tissues sections were photographed under a light microscope (Leica DM4 P, Germany) to determine anatomical parameters.

### 4.4. Protein Extraction and Quantification

Protein extraction was according to the method described by He and Wang [59] with some modifications. The 2–4 g of treated tissue from *A. ilicifolius* and *A. mollis* were ground in liquid nitrogen and extracted using the tricarboxylic acid (TCA)-acetone/phenol-methanol combined extraction method. The tissue powder was transferred to a centrifuge tube and precipitated by adding cold acetone solution containing 0.2% dithiothreitol (DTT), then the supernatant was discarded and the pellet was suspended in the 2× extraction buffer (20 mmol·L^−1^ Tris-HCl (pH 8.0), 250 mmol·L^−1^ sucrose, 10 mmol·L^−1^ ethylene glycol tetraacetic acid, 1 mmol·L^−1^ phenylmethylsulfonyl fluoride, 1% Triton X-100, 2% β-mercaptoethanol) at 4 °C for 15 min. The homogenate was adding an equal volume of saturated phenol (pH 7.5) to obtain the upper phenol phase, then mixed with three volumes of ice-cold methanol (containing 0.1 mol·L^−1^ ammonium acetate) overnight at −20 °C. Then the pellet was washed with ammonium acetate/methanol (0.1 mol·L^−1^) and acetone (containing 0.2% DTT). Protein quantification was performed using the Bradford [60] method with bicinchoninic acid as the standard. Each treatment was performed for three biological replications for quantitative analysis.

An immobiline dry strip gel (17 cm, pH 4–7; Bio-Rad Laboratories, Inc., Hercules, CA, USA) and 12.5% polyacrylamide gels were used to separate the prepared samples in the first and second dimension, respectively. The sodium dodecyl sulfate-polyacrylamide gel electrophoresis (SDS-PAGE) gels were stained with Coomassie Brilliant Blue R-250 and scanned with Uniscan M3600 (China) at 600 dpi. Gel alignment, spots detection and quantification were done using PDQuest software (Version 8.0, Bio-Rad). The DEPs were obtained by pairwise comparison with a fold change ≥ 2.0 and a Student’s t-test (*p* < 0.05). In-gel tryptic digestion and protein identification were performed according to the method of Liu et al. [61].

### 4.5. Determinations of AMP, ADP, ATP, and Sugar Content

The method of ATP, ADP, and AMP extraction was according to Chen et al. [62] with some modifications. The powder was obtained from 2 g tissue and then homogenized with 10 mL perchloric acid (0.6 mol·L^−1^) at 4 °C for 30 min, then centrifuged at 6000 rpm for 20 min. The resulting supernatant (6 mL) was quickly neutralized to pH 6.5–6.8 with 1 mol·L^−1^ potassium hydroxide solution and passed through a 0.22 μm syringe filter. The supernatant was diluted to 10 mL before measuring. Shimadzu^®^ LC-20A Prominence high-performance liquid chromatography (HPLC) equipped with Syncronis C18 column (4.6 mm × 250 mm, Thermo Fisher Scientific) was used to measure ATP, ADP, and AMP contents. The mobile phase was 0.1% phosphoric acid and the flow rate was 0.8 mL·min^−1^. The ultraviolet detection wavelength was 254 nm. The energy charge (EC) was calculated using the following formula: EC = ([ATP] + 1/2 [ADP])/([ATP] + [ADP] + [AMP]) [62]. Data were expressed as means of the five replicates.

The starch and total soluble sugar contents were measured with the starch content kit and plant soluble sugar content test kit, respectively (Nanjing Jian Cheng Institute, Nanjing, China).

### 4.6. Data Analysis

The experimental data were evaluated with IBM SPSS Statistics for Mac (Version 23.0). Principal component analysis (PCA) was performed using OriginPro 2021 (OriginLab Corp, Northampton, MA, USA). The classification of identified proteins was performed using the UniProt Knowledgebase (http://www.uniprot.org) and the NCBI (https://www.ncbi.nlm.nih.gov). The PPI network analysis was acquired using the Search Tool for the Retrieval of Interacting Genes/Proteins (STRING) version 11.0 (https://string-db.org). Cytoscape software of the PPI network can visualize significant protein-protein associations [63]. The Cytoscape plugin (cytoHubba) was utilized to evaluate hub proteins from the PPI network by Matthews correlation coefficient (MCC) method [63]. We used the degree score to identify hub proteins.

## 5. Conclusions

**Figure 9:**
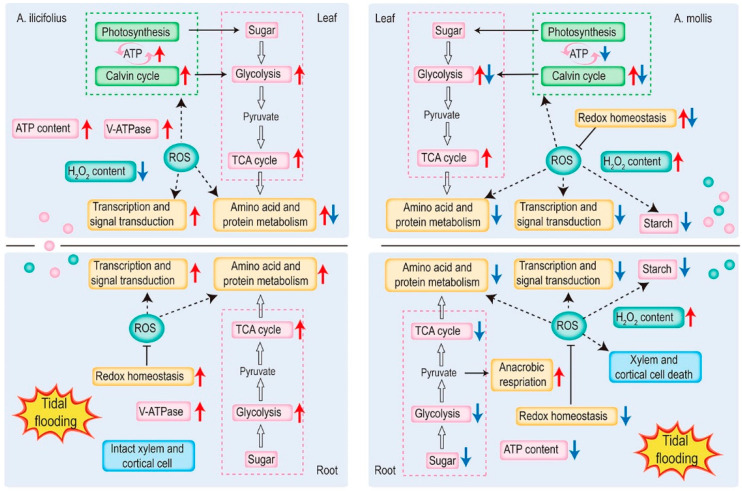
A schematic representation of *A. ilicifolius* and *A. mollis* response and adaptation strategies to tidal flooding stress. Red arrows, up-regulated; blue arrows, down-regulated; pink, the energy-producing pathway and storage substance; orange, biological metabolism that requires energy; green, the pathways in chloroplast. Pink circle, the transport of energy and sugar from leaves to roots; cyan circle, the transport of H_2_O_2_.

Physiological and proteomic analyses have greatly enriched the current knowledge of flooding resistance in Acanthus species. *A. ilicifolius* performed better under tidal flooding stress, which was reflected in the integrity of the morphological structure, a high level of energy charge, and an increase in the ratio of soluble sugar to starch. A higher percentage of up-regulated proteins associated with carbon and energy metabolism were found in *A. ilicifolius* tissues under tidal flooding stress. However, the change in the root structure was not responsible for adaption to flooding conditions at the early stage and the maintenance of physiological homeostasis had higher demands for essential supply of energy. *A. mollis* leaves remained structurally intact even after tidal flooding stress, which might be due to partially enhanced ROS scavenging capacity and carbon and energy metabolism. The disruption of energy provision and flux balance in *A. mollis* roots demonstrates that maintenance of an energy balance under abiotic stress is critical for cell survival. As shown in Figure 9, we propose a working model to illustrate the detailed mechanism of *A. ilicifolius* and *A. mollis* under tidal flooding stress.

## Data Availability

Not applicable.

## Supplementary Materials

Supplementary Materials can be found at https://www.mdpi.com/1422-0067/22/3/1055/s1.
