# Supplementary material for: Physiological and Proteomic Analyses of Two Acanthus Species to Tidal Flooding Stress
Source: Int J Mol Sci. 2021 Jan 21;22(3):1055. doi: 10.3390/ijms22031055 (PMC7865619; doi:10.3390/ijms22031055)
Supplement: Supplementary file 1 [file ijms-22-01055-s001.zip › Supplementary Figure S1-S4.docx]

**Supplementary**


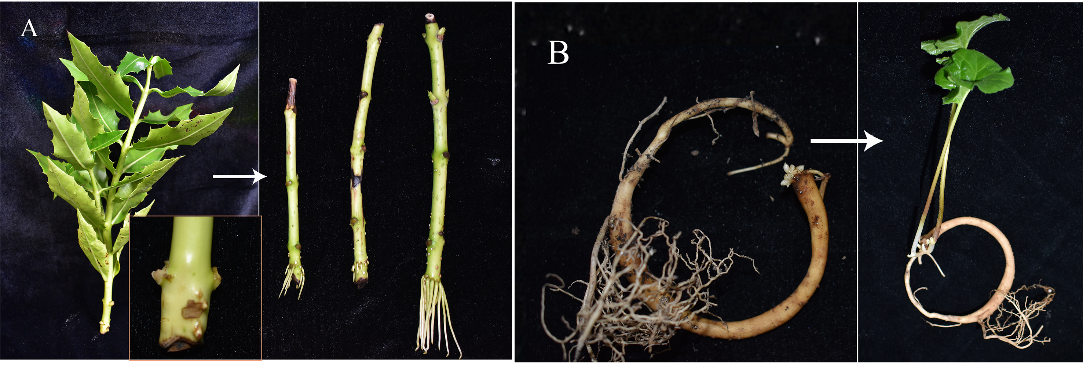


Supplementary Fig. S1. The asexual reproduction of (A) *A. ilicifolius* and (B) *A. mollis*.


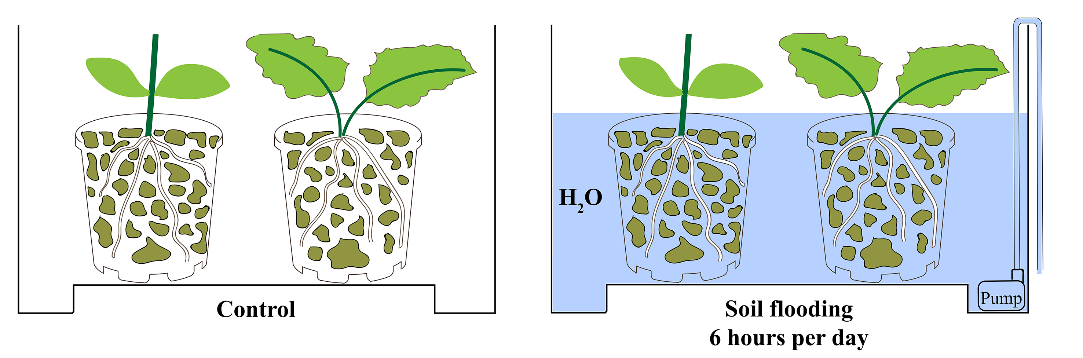


Supplementary Fig. S2. Diagram illustrating experimental set up.


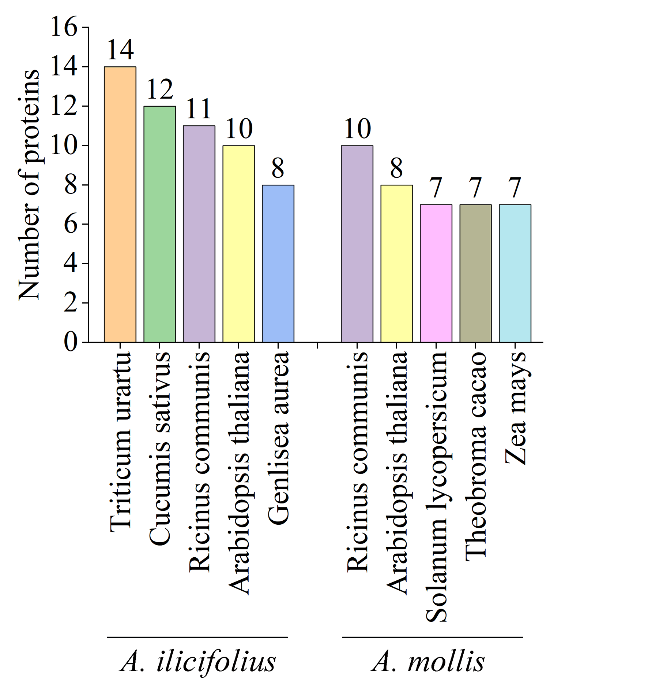


Supplementary Fig. S3. Top-five species that has high homology with the identiﬁed protein in the tissues of *A. ilicifolius* and *A. mollis*.


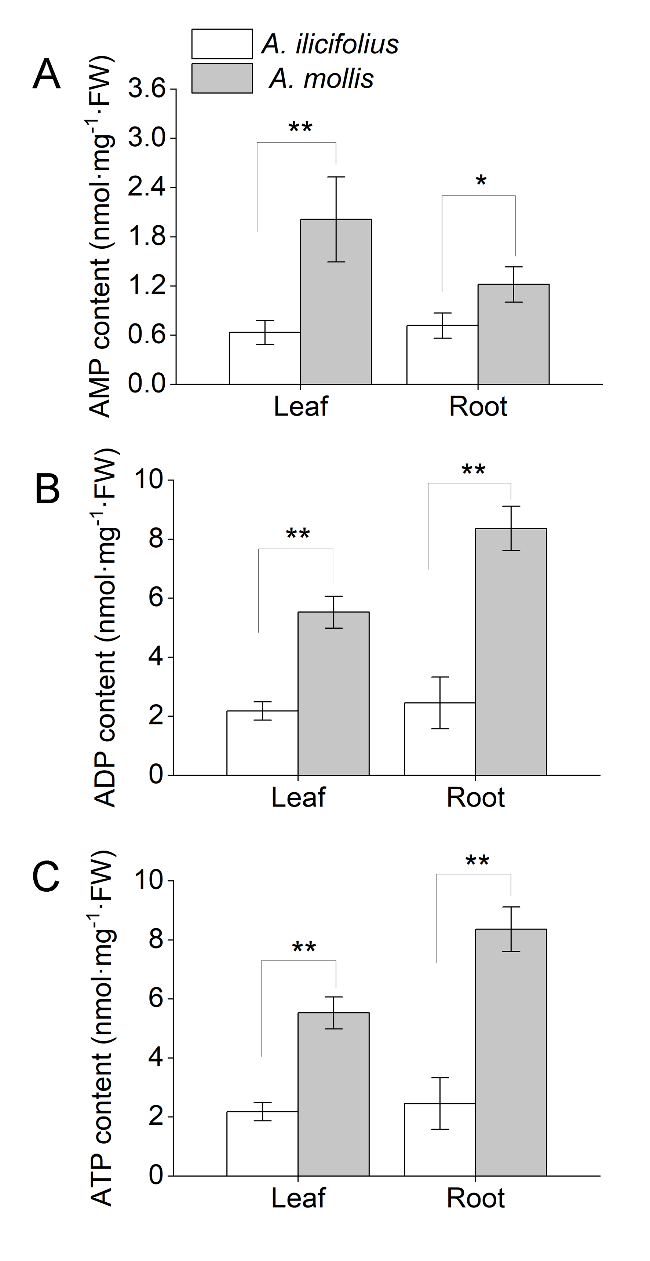


Supplementary Fig. S4. (A) AMP content, (B) ADP content, and (C) ATP content of *A. ilicifolius* and *A. mollis* in the control group*.* * and ** indicate significant difference at the 0.05 level and the 0.01 level, respectively.
