# Supplementary material for: Physiological and Proteomic Analyses of Two Acanthus Species to Tidal Flooding Stress
Source: Int J Mol Sci. 2021 Jan 21;22(3):1055. doi: 10.3390/ijms22031055 (PMC7865619; doi:10.3390/ijms22031055)
Supplement: Supplementary file 1 [file ijms-22-01055-s001.zip › Supplementary Table S1-5,7.docx]

**Supplementary Table S1.** Relative plant dry weight of *A. ilicifolius* and *A. mollis*.

| Tissue | Species | Tidal flooding (days) | | | | |
| --- | --- | --- | --- | --- | --- | --- |
|  |  | 0 | 4 | 8 | 10 | 12 |
| Leaf | *A. ilicifolius* | 101.079±10.460 a | 96.172±11.273 a | 97.172±14.879 a | 103.838±19.334 a | 102.839±17.830 a |
|  | *A. mollis* | 106.903±19.600 a | 107.017±9.615 a | 83.888±11.665 b | 85.451±14.898 b | 74.621±12.257 b |
| Root | *A. ilicifolius* | 99.777±13.026 a | 89.615±12.844 a | 108.087±19.528 a | 115.356±17.629 a | 96.395±32.824 a |
|  | *A. mollis* | 111.014±22.324 a | 82.603±5.970 a | 57.512±5.849 b | 49.281±8.053 b | 45.249±7.897 b |

**Supplementary Table 2.** Identification of DEPs of *A. ilicifolius* leaves with an expression change greater than 2.0-fold change under tidal flooding stress

| Spot ^a^ | Accession (gb) ^b^ | Protein Name ^c^ | Thero. ^d^ kDa/p*I* | Exper. ^e^ kDa/p*I* | Score^f^ | MP ^g^ | Species ^h^ | SF vs. CK ^i^ |
| --- | --- | --- | --- | --- | --- | --- | --- | --- |
| Photosynthesis and photorespiration | | |  |  |  |  |  |  |
| 1 | gi\|222842405 | Plastocyanin family protein | 17.07/4.94 | 6.80/4.00 | 98 | 2 | *Populus trichocarpa* | -6.268 |
| 2 | gi\|449515811 | Predicted: chlorophyll a-b binding protein 40, chloroplastic-like, partial | 15.98/6.58 | 18.87/4.93 | 88 | 2 | *Cucumis sativus* | -1.555 |
| 12 | gi\|222859802 | Chlorophyll a-b binding protein 2 | 28.09/5.29 | 15.44/5.07 | 87 | 6 | *Populus trichocarpa* | 1.937 |
| 13 | gi\|475542040 | Chlorophyll a-b binding protein, chloroplastic | 28.72/5.14 | 19.47/4.74 | 114 | 6 | *Aegilops tauschii* | 3.202 |
| 16 | gi\|449442663 | Predicted: phosphoglycolate phosphatase-like | 41.72/6.47 | 24.00/4.78 | 63 | 3 | *Cucumis sativus* | 3.455 |
| 18 | gi\|474352688 | Oxygen-evolving enhancer protein 1, chloroplastic | 34.64/5.75 | 36.97/4.93 | 352 | 7 | *Triticum urartu* | 2.893 |
| 33 | gi\|223540996 | Chlorophyll a/b binding protein, putative | 31.10/5.52 | 16.37/5.43 | 75 | 5 | *Ricinus communis* | -1.887 |
| 58 | gi\|428230860 | Chlorophyll binding protein, partial | 21.39/5.19 | 15.41/5.93 | 66 | 2 | *Clermontia arborescens* subsp. *Waihiae* | 6.846 |
| 65 | gi\|475616276 | Putative quinone-oxidoreductase-like protein, chloroplastic | 35.43/9.13 | 27.96/5.86 | 69 | 11 | *Aegilops tauschii* | -2.415 |
| 71 | gi\|550338673 | Chain A family protein | 40.79/8.54 | 23.76/6.45 | 70 | 10 | *Populus trichocarpa* | -6.268 |
| Calvin cycle | | |  |  |  |  |  |  |
| 4 | gi\|475522663 | Ribulose bisphosphate carboxylase/oxygenase activase B, chloroplastic | 51.47/8.86 | 38.41/4.86 | 268 | 5 | *Aegilops tauschii* | -5.046 |
| 5 | gi\|508726181 | Rubisco activase isoform 2 | 52.37/5.26 | 37.73/4.91 | 407 | 10 | *Theobroma cacao* | 1.103 |
| 7 | gi\|508787184 | RuBisCO large subunit-binding protein subunit alpha isoform 1 | 64.07/5.06 | 58.37/4.67 | 108 | 9 | *Theobroma cacao* | 1.518 |
| 9 | gi\|502125499 | Predicted: ruBisCO large subunit-binding protein subunit beta, chloroplastic-like | 63.20/5.85 | 52.72/5.05 | 162 | 6 | *Cicer arietinum* | 5.431 |
| 15 | gi\|542718032 | Ribulose-1,5-bisphosphate carboxylase/oxygenase large subunit, partial (chloroplast) | 38.09/6.49 | 25.25/5.22 | 294 | 11 | *Prunus wilsoni* | 3.362 |
| 19 | gi\|410927414 | Chloroplast ribulose bisphosphate carboxylase/oxygenase activase beta1, partial | 33.16/5.09 | 32.55/5.14 | 274 | 4 | *Gossypium barbadense* | 1.568 |
| 39 | gi\|223541989 | Phosphoribulose kinase, putative | 45.22/5.83 | 30.46/5.31 | 302 | 11 | *Ricinus communis* | 2.349 |
| 51 | gi\|399139809 | Ribulose-1,5-bisphosphate carboxylase/oxygenase large subunit, partial | 51.58/6.30 | 20.22/6.70 | 388 | 9 | *Strobilanthes glutinosus* | -3.465 |
| 61 | gi\|406366546 | Ribulose-1,5-bisphosphate carboxylase/oxygenase small subunit, partial (chloroplast) | 19.30/8.80 | 23.30/6.17 | 61 | 8 | *Gossypium gossypioides* | 1.000 |
| Carbon metabolism | |  |  |  |  |  |  |  |
| 34 | gi\|449458564 | Predicted: triosephosphate isomerase, chloroplastic-like | 33.00/7.01 | 17.22/5.56 | 81 | 4 | *Cucumis sativus* | 1.349 |
| 38 | gi\|317373797 | Chloroplast phosphoglycerate kinase 3 | 50.28/6.69 | 32.06/5.55 | 121 | 10 | *Helianthus annuus* | 1.671 |
| 46 | gi\|527186354 | Triosephosphate isomerase | 33.67/6.90 | 16.94/5.83 | 314 | 6 | *Genlisea aurea* | 3.564 |
| 48 | gi\|332005925 | 6-phosphogluconolactonase 4 | 29.51/6.23 | 17.67/5.40 | 91 | 2 | *Arabidopsis thaliana* | 2.677 |
| 49 | gi\|222864107 | Cytosolic phosphoglycerate kinase family protein | 42.77/5.70 | 30.36/5.89 | 314 | 7 | *Populus trichocarpa* | 2.056 |
| 52 | gi\|223547261 | Phosphoglycerate kinase, putative | 50.11/8.74 | 35.17/5.64 | 95 | 2 | *Ricinus communis* | 1.764 |
| 56 | gi\|350538295 | Enolase | 48.05/5.68 | 46.18/5.87 | 233 | 8 | *Solanum lycopersicum* | 2.329 |
| 68 | gi\|485820030 | Enolase, partial | 11.86/7.98 | 41.86/5.95 | 84 | 3 | *Schiedea heller* | 2.103 |
| 73 | gi\|508711124 | Glyceraldehyde-3-phosphate dehydrogenase A subunit2 | 43.26/8.15 | 35.69/6.73 | 407 | 11 | *Theobroma cacao* | -2.508 |
| 75 | gi\|413916139 | Glycine cleavage complex P-protein | 119.11/6.79 | 88.83/6.36 | 118 | 8 | *Zea mays* | 2.887 |
| 76 | gi\|413916139 | Glycine cleavage complex P-protein | 119.11/6.79 | 89.61/6.42 | 99 | 8 | *Zea mays* | 3.691 |
| TCA cycle | |  |  |  |  |  |  |  |
| 63 | gi\|332189573 | Malate dehydrogenase | 35.89/6.11 | 28.43/5.94 | 70 | 7 | *Arabidopsis thaliana* | 1.083 |
| 64 | gi\|223526678 | Malate dehydrogenase, putative | 35.98/6.40 | 28.39/6.19 | 183 | 2 | *Ricinus communis* | 1.026 |
| 74 | gi\|499138229 | Dihydrolipoamide dehydrogenase, partial | 43.94/7.79 | 50.17/6.64 | 145 | 4 | *Rhizophora stylosa* | 1.362 |
| Energy metabolism | | |  |  |  |  |  |  |
| 6 | gi\|449454235 | Predicted: V-type proton ATPase subunit B 1-like | 54.36/4.96 | 47.50/4.88 | 103 | 10 | *Cucumis sativus* | 1.704 |
| 21 | gi\|545719412 | ATP synthase CF1 alpha subunit (chloroplast) | 55.57/5.15 | 48.13/5.10 | 547 | 18 | *Allosyncarpia ternata* | 3.568 |
| 23 | gi\|393396089 | ATP synthase CF1 beta subunit (chloroplast) | 53.80/5.20 | 44.61/5.25 | 774 | 18 | *Vigna unguiculata* | 1.176 |
| 24 | gi\|449434570 | Predicted: ATP synthase subunit beta, mitochondrial-like | 59.89/5.90 | 45.30/5.29 | 571 | 16 | *Cucumis sativus* | -1.176 |
| 32 | gi\|508726652 | ATP synthase D chain, mitochondrial | 19.65/0.00 | 11.60/5.35 | 98 | 5 | *Theobroma cacao* | -4.169 |
| 43 | gi\|350537279 | Vacuolar H^+^-ATPase A2 subunit isoform | 68.96/5.30 | 58.53/5.43 | 534 | 19 | *Solanum lycopersicum* | 1.026 |
| 44 | gi\|350537129 | Vacuolar H^+^-ATPase A1 subunit isoform | 68.81/5.20 | 60.95/5.35 | 268 | 14 | *Solanum lycopersicum* | 3.239 |
| 45 | gi\|223550217 | ATP-dependent clp protease, putative | 103.12/6.27 | 73.67/5.53 | 382 | 19 | *Ricinus communis* | 1.578 |
| 50 | gi\|222848536 | ATP synthase gamma chain 1 family protein | 41.36/8.16 | 26.16/5.92 | 263 | 6 | *Populus trichocarpa* | 1.438 |
| Amino acid and protein metabolism | | |  |  |  |  |  |  |
| 8 | gi\|449442347 | Predicted: stromal 70 kDa heat shock-related protein, chloroplastic-like | 75.46/5.18 | 70.85/4.68 | 67 | 7 | *Cucumis sativus* | 2.747 |
| 10 | gi\|332003097 | Heat shock cognate protein 70-1 | 71.71/5.03 | 64.76/5.05 | 322 | 16 | *Arabidopsis thaliana* | 4.142 |
| 11 | gi\|460369188 | Predicted: stromal 70 kDa heat shock-related protein, chloroplastic-like | 74.96/5.20 | 72.88/4.79 | 97 | 3 | *Solanum lycopersicum* | 2.282 |
| 17 | gi\|508722909 | 20S proteasome alpha subunit F2 | 33.23/4.76 | 28.45/5.08 | 120 | 4 | *Theobroma cacao* | 2.319 |
| 25 | gi\|508784980 | TCP-1/cpn60 chaperonin family protein | 64.51/5.62 | 50.84/5.08 | 374 | 6 | *Theobroma cacao* | -2.677 |
| 26 | gi\|508726275 | Heat shock cognate protein 70-1 | 71.75/5.03 | 64.94/5.09 | 646 | 19 | *Theobroma cacao* | 1.306 |
| 27 | gi\|508784980 | TCP-1/cpn60 chaperonin family protein | 64.51/5.62 | 50.98/5.12 | 503 | 15 | *Theobroma cacao* | -2.093 |
| 28 | gi\|293334615 | Heat shock cognate 70 kDa protein 2 | 71.52/5.13 | 64.16/5.13 | 329 | 13 | *Zea mays* | 1.565 |
| 29 | gi\|332643321 | Heat shock protein 60 | 61.58/5.66 | 52.08/5.21 | 163 | 7 | *Arabidopsis thaliana* | 6.843 |
| 35 | gi\|223544718 | Groes chaperonin, putative | 26.58/8.89 | 17.09/5.10 | 61 | 5 | *Ricinus communis* | -3.754 |
| 37 | gi\|425856442 | Mta/sah nucleosidase, partial | 23.86/5.67 | 29.25/5.46 | 69 | 2 | *Galium verum var. asiaticum* | 1.668 |
| 53 | gi\|343465772 | Plastid glutamine synthetase isoform | 47.02/5.75 | 34.37/5.76 | 182 | 7 | *Secale cereale x Triticum durum* | 2.960 |
| 54 | gi\|332642427 | S-adenosyethionine synthase 4 | 43.17/5.51 | 37.04/5.81 | 193 | 8 | *Arabidopsis thaliana* | -1.269 |
| 55 | gi\|475453557 | 26S protease regulatory subunit 6B-like protein | 30.13/6.54 | 46.00/5.57 | 155 | 9 | *Aegilops tauschii* | -1.781 |
| 57 | gi\|475603792 | Heat shock 70 kDa protein, mitochondrial | 72.91/5.53 | 58.32/5.58 | 113 | 5 | *Aegilops tauschii* | -2.900 |
| 59 | gi\|332646593 | Proteasome subunit beta type-1 | 24.86/6.95 | 16.99/6.04 | 149 | 4 | *Arabidopsis thaliana* | 2.448 |
| 66 | gi\|550319185 | Glutamate-ammonia ligase family protein | 39.42/5.95 | 32.83/6.07 | 153 | 7 | *Populus trichocarpa* | 1.970 |
| 67 | gi\|332642304 | Mitochondrial processing peptidase alpha subunit | 54.19/6.04 | 39.89/6.12 | 104 | 2 | *Arabidopsis thaliana* | 1.262 |
| 69 | gi\|449450860 | Elongation factor 2-like | 95.03/5.97 | 86.42/6.25 | 133 | 8 | *Cucumis sativus* | -3.222 |
| 77 | gi\|226491656 | Peptidyl-prolyl cis-trans isomerase | 26.37/0.00 | 10.97/6.84 | 155 | 6 | *Zea mays* | -1.714 |
| 78 | gi\|543177006 | Peptidyl-prolyl cis-trans isomerase | 27.37/9.46 | 17.33/6.99 | 277 | 4 | *Phaseolus vulgaris* | -5.867 |
| Stress and defense | | |  |  |  |  |  |  |
| 36 | gi\|474311703 | L-ascorbate peroxidase 1, cytosolic | 27.56/5.85 | 18.92/5.45 | 117 | 5 | *Triticum urartu* | 1.505 |
| 70 | gi\|436805717 | Copper/zinc-superoxide dismutase | 15.39/5.47 | 8.88/6.22 | 96 | 2 | *Litchi chinensis* | -6.172 |
| Transcription and signal transduction | | |  |  |  |  |  |  |
| 3 | gi\|26454609 | 14-3-3 protein 7 | 28.91/4.96 | 22.71/4.65 | 137 | 3 | *Solanum lycopersicum* | 2.365 |
| 14 | gi\|449469841 | Predicted: 14-3-3-like protein-like | 29.64/4.77 | 19.79/4.67 | 90 | 3 | *Cucumis sativus* | 1.661 |
| 22 | gi\|508718683 | Tubulin alpha-5 | 54.00/4.98 | 44.65/5.20 | 433 | 10 | *Theobroma cacao* | 1.983 |
| 30 | gi\|449464210 | Predicted: leukotriene A-4 hydrolase homolog | 69.93/5.37 | 67.72/4.88 | 157 | 5 | *Cucumis sativus* | 1.890 |
| 31 | gi\|508782306 | Eukaryotic translation initiation factor 5A-1 | 17.77/5.60 | 10.09/5.55 | 115 | 3 | *Theobroma cacao* | 1.953 |
| 40 | gi\|386278562 | Actin7a, partial | 39.39/5.21 | 36.73/5.45 | 271 | 9 | *Vernicia fordii* | 1.136 |
| 41 | gi\|223540420 | Cell division protein ftsH, putative | 75.50/6.43 | 57.28/5.21 | 467 | 7 | *Ricinus communis* | 2.854 |
| 42 | gi\|475605012 | Cell division protease ftsH-like protein, chloroplastic | 71.94/5.60 | 57.89/5.09 | 271 | 8 | *Aegilops tauschii* | 2.976 |
| 47 | gi\|15237579 | RNA-binding protein NOB1 | 67.08/5.55 | 16.04/5.67 | 61 | 14 | *Arabidopsis thaliana* | 2.558 |
| 62 | gi\|355477483 | F-box family protein | 19.67/4.56 | 18.75/6.20 | 63 | 2 | *Medicago truncatula* | -1.196 |
| 72 | gi\|514725733 | Predicted: chloroplast stem-loop binding protein of 41 kDa, chloroplastic-like | 41.49/6.41 | 31.25/6.73 | 203 | 6 | *Setaria italica* | -1.133 |
| Others proteins | | |  |  |  |  |  |  |
| 20 | gi\|508727025 | Phosphate transporter traffic facilitator isoform 2 | 34.31/6.46 | 46.38/5.08 | 62 | 6 | *Theobroma cacao* | 4.787 |
| 60 | gi\|502120213 | Predicted: flocculation protein FLO11-like isoform X2 | 66.28/10.59 | 18.40/5.99 | 69 | 4 | *Cicer arietinum* | 5.023 |

^a^ The spot number corresponding to the number listed in the supplementary table 2.

^b^ Database accession numbers (gb) according to NCBInr.

^c^ The name of proteins was identiﬁed by LC-MALDI-TOF/TOF.

^d^ Theoretical mass (kDa) and p*I* of identiﬁed proteins. Theoretical values were retrieved from the NCBInr database.

^e^ Experimental mass (kDa) and p*I* of identiﬁed proteins. Experimental values were calculated by using PDquest software and standard molecular mass markers.

^f^ The Mascot searched score against the database NCBInr.

^g^ Number of matched peptide fragments.

^h^ The species that has the high homology of the identiﬁed protein.

^i^ Log_2_- (fold change) values between the different treatments. SF vs CK means soil flooding treatment vs control group.

**Supplementary Table** **3.** Identiﬁcation of DEPs of *A. ilicifolius* roots with an expression change greater than 2.0-fold change under tidal flooding stress

| Spot ^a^ | Accession (gb)^b^ | Protein Name ^c^ | Thero. ^d^ kDa/p*I* | Exper. ^e^ kDa/p*I* | Score^f^ | MP ^g^ | Species ^h^ | SF vs. CK ^i^ |
| --- | --- | --- | --- | --- | --- | --- | --- | --- |
| TCA cycle | | |  |  |  |  |  |  |
| R37 | gi\|226503019 | Malate dehydrogenase, cytoplasmic | 35.84/5.76 | 13.42/5.98 | 127 | 2 | *Zea mays* | 1.359 |
| Carbon and energy metabolism | | |  |  |  |  |  |  |
| R10 | gi\|460407876 | V-type proton ATPase subunit d2-like | 41.3/4.9 | 36.53/4.62 | 154 | 6 | *Solanum lycopersicum* | -3.684 |
| R11 | gi\|449454235 | Predicted: V-type proton ATPase subunit B 1-like | 54.36/4.96 | 52.22/4.67 | 393 | 9 | *Cucumis sativus* | 6.056 |
| R12 | gi\|470108902 | Predicted: V-type proton ATPase subunit B2-like | 54.61/5.07 | 53.04/4.70 | 72 | 8 | *Fragaria vesca* subsp. *vesca* | 4.146 |
| R16 | gi\|490262869 | ATP synthase subunit D, partial | 19.24/5.21 | 14.44/5.18 | 102 | 2 | *Hydnora visseri* | 1.438 |
| R19 | gi\|355479515 | Adenosine kinase | 38.08/5.08 | 33.59/5.14 | 104 | 4 | *Medicago truncatula* | 3.827 |
| R22 | gi\|473798701 | ATP synthase subunit beta, mitochondrial | 57.83/5.25 | 49.52/5.15 | 719 | 10 | *Triticum urartu* | 1.402 |
| R26 | gi\|346683384 | ATPase subunit 1 | 55.24/5.58 | 61.31/6.16 | 435 | 11 | *Cucumis sativus* | 2.206 |
| R29 | gi\|449434570 | Predicted: ATP synthase subunit beta, mitochondrial-like | 59.89/0.00 | 49.56/5.27 | 121 | 16 | *Cucumis sativus* | 5.272 |
| R30 | gi\|110288667 | Enolase, putative, expressed | 51.89/5.72 | 52.95/5.29 | 492 | 11 | *Oryza sativa* Japonica Group | 1.581 |
| R32 | gi\|398363571 | Fructokinase | 34.69/5.49 | 28.6/5.46 | 123 | 4 | *Actinidia deliciosa* | 3.000 |
| R38 | gi\|527196189 | Nucleoside diphosphate kinase | 16.48/6.43 | 52.61/6.17 | 143 | 4 | *Genlisea aurea* | 2.445 |
| Amino acid and protein metabolism | | |  |  |  |  |  |  |
| R7 | gi\|223532621 | Proteasome subunit beta type 6,9, putative | 24.91/5.17 | 18.50/4.78 | 109 | 3 | *Ricinus communis* | -4.315 |
| R13 | gi\|315307966 | Heat shock protein 90-1 | 80.45/4.96 | 67.92/4.85 | 155 | 5 | *Nicotiana attenuata* | 5.239 |
| R14 | gi\|527187624 | Heat shock protein 70 | 71.62/5.06 | 67.43/4.92 | 513 | 22 | *Genlisea aurea* | 8.424 |
| R15 | gi\|223535705 | 60S ribosomal protein L23, putative | 7.17/11.09 | 11.34/5.12 | 74 | 4 | *Ricinus communis* | 4.451 |
| R23 | gi\|508784980 | TCP-1/cpn60 chaperonin family protein | 64.51/5.62 | 56.16/4.98 | 159 | 6 | *Theobroma cacao* | 2.581 |
| R27 | gi\|527189531 | Protein disulfide-isomerase, partial | 38.13/5.26 | 31.65/5.33 | 109 | 4 | *Genlisea aurea* | 4.561 |
| R33 | gi\|332656685 | S-adenosyethionine synthase 2 | 43.63/5.67 | 44.43/5.56 | 346 | 7 | *Arabidopsis thaliana* | 2.857 |
| R34 | gi\|351722651 | Glutamine synthetase cytosolic isozyme 1 | 38.99/5.46 | 32.95/5.74 | 154 | 5 | *Glycine max* | 7.338 |
| R40 | gi\|351722651 | Glutamine synthetase cytosolic isozyme 1 | 38.99/5.46 | 25.31/6.17 | 186 | 6 | *Glycine max* | 3.97 |
| Stress and defense | | |  |  |  |  |  |  |
| R1 | gi\|223529085 | Peroxidase, putative | 19.45/8.6 | 32.03/4.05 | 74 | 1 | *Ricinus communis* | -1.056 |
| R17 | gi\|474311703 | L-ascorbate peroxidase 1, cytosolic | 27.56/5.85 | 23.23/5.14 | 160 | 4 | *Triticum urartu* | 1.219 |
| R20 | gi\|527187175 | Monodehydroascorbate reductase | 47.2/5.82 | 39.32/5.12 | 118 | 3 | *Genlisea aurea* | 4.013 |
| R31 | gi\|427199300 | Thioredoxin | 13.67/5.76 | 11.16/5.73 | 183 | 4 | *Ipomoea batatas* | 1.824 |
| R36 | gi\|223551378 | Catalase, putative | 113.36/6.84 | 38.22/5.88 | 234 | 11 | *Ricinus communis* | 3.83 |
| Transcription and signal transduction | | |  |  |  |  |  |  |
| R2 | gi\|526117762 | 14-3-3 protein | 29.47/4.79 | 23.65/4.48 | 132 | 2 | *Vitis vinifera* | 1.661 |
| R3 | gi\|350539221 | 14-3-3 protein 7 | 28.91/4.96 | 24.95/4.33 | 110 | 2 | *Solanum lycopersicum* | 1.531 |
| R4 | gi\|543176851 | 14-3-3 protein | 29.26/4.66 | 24.57/4.41 | 277 | 7 | *Phaseolus vulgaris* | -1.475 |
| R21 | gi\|527203530 | Actin-97 | 41.95/5.37 | 41.35/5.15 | 698 | 14 | *Genlisea aurea* | 2.133 |
| R24 | gi\|473749533 | NuA3 HAT complex component NTO1 | 103.46/7.88 | 12.9/5.36 | 63 | 17 | *Triticum urartu* | 4.488 |
| R25 | gi\|375968572 | SKP1 protein | 17.63/0.00 | 14.72/5.37 | 141 | 5 | *Nicotiana tabacum* | -1.983 |
| R28 | gi\|527203530 | Actin-97 | 41.95/5.37 | 41.29/5.26 | 706 | 16 | *Genlisea aurea* | 3.076 |
| Other proteins | | |  |  |  |  |  |  |
| R8 | gi\|508724744 | Pathogen-related protein | 27.54/5.10 | 25.65/4.76 | 64 | 4 | *Theobroma cacao* | -1.063 |
| R39 | gi\|15239652 | Flavodoxin-like quinone reductase 1 | 21.40/5.96 | 15.6/6.43 | 84 | 2 | *Arabidopsis thaliana* | 4.289 |
| Unknown proteins | |  |  |  |  |  |  |  |
| R5 | gi\|125562472 | Hypothetical protein OsI_30174 | 16.21/8.51 | 59.29/4.46 | 73 | 6 | *Oryza sativa* Indica Group | 5.139 |
| R6 | gi\|226521422 | Predicted protein | 88.7/5.56 | 59.7/4.49 | 68 | 14 | *Micromonas sp. RCC299* | 3.272 |
| R9 | gi\|527206839 | Hypothetical protein M569_02468 | 27.34/9.01 | 32.97/4.88 | 76 | 4 | *Genlisea aurea* | -4.319 |
| R18 | gi\|557113120 | Hypothetical protein EUTSA_v10025711mg | 34.88/6.07 | 28.49/5.14 | 139 | 4 | *Eutrema salsugineum* | -1.621 |
| R35 | gi\|557531169 | Hypothetical protein CICLE_v10012166mg | 36.16/9.43 | 25.17/5.9 | 140 | 3 | *Citrus clementina* | -3.594 |

^a^ The spot number corresponding to the number listed in the supplementary table 3. R represents the root tissue*.*

^b^ Database accession numbers (gb) according to NCBInr.

^c^ The name of proteins was identiﬁed by LC-MALDI-TOF/TOF.

^d^ Theoretical mass (kDa) and p*I* of identiﬁed proteins. Theoretical values were retrieved from the NCBInr database.

^e^ Experimental mass (kDa) and p*I* of identiﬁed proteins. Experimental values were calculated by using PDquest software and standard molecular mass markers.

^f^ The Mascot searched score against the database NCBInr.

^g^ Number of matched peptide fragments.

^h^ The species that has the high homology of the identiﬁed protein.

^i^ Log_2_- (fold change) values between the different treatments. SF vs CK means soil flooding treatment vs control group.

**Supplementary Table** **4.** Identiﬁcation of DEPs of *A. mollis* leaves with an expression change greater than 2.0-fold change under tidal flooding stress

| Spot ^a^ | Accession (gb) ^b^ | Protein Name ^c^ | Thero. ^d^ kDa/p*I* | Exper. ^e^ kDa/p*I* | Score^f^ | MP ^g^ | Species ^h^ | SF vs. CK ^i^ |
| --- | --- | --- | --- | --- | --- | --- | --- | --- |
| Photosynthesis and photorespiration | | |  |  |  |  |  |  |
| 20 | gi\|543176923 | Oxygen-evolving enhancer protein 1 | 35.20/6.08 | 19.74/5.42 | 170 | 4 | *Phaseolus vulgaris* | -2.242 |
| 26 | gi\|474445723 | Phosphoglycolate phosphatase | 33.22/8.87 | 24.64/5.41 | 64 | 4 | *Triticum urartu* | -1.575 |
| 33 | gi\|223539254 | Oxygen-evolving enhancer protein 2, chloroplast precursor, putative | 28.76/8.63 | 13.68/5.79 | 130 | 4 | *Ricinus communis* | -2.109 |
| 43 | gi\|474121685 | Chlorophyll a-b binding protein 8, chloroplastic | 29.29/8.69 | 11.86/5.78 | 109 | 3 | *Triticum urartu* | -2.066 |
| 52 | gi\|223551247 | Ferredoxin-NADP reductase, putative | 40.74/8.70 | 14.35/6.50 | 69 | 9 | *Ricinus communis* | 4.265 |
| 63 | gi\|527197786 | Cytochrome b6-f complex iron-sulfur subunit 1, chloroplastic, partial | 24.25/8.48 | 11.51/6.78 | 1.9 | 4 | *Genlisea aurea* | 2.970 |
| 64 | gi\|330318806 | Photosystem I reaction center subunit iv b | 11.37/9.88 | 12.76/6.95 | 67 | 4 | *Camellia sinensis* | 2.491 |
| Calvin cycle | |  |  |  |  |  |  |  |
| 3 | gi\|399139356 | Ribulose-1,5-bisphosphate carboxylase/oxygenase large subunit, partial | 51.49/6.46 | 34.61/4.81 | 654 | 19 | *Anisochilus pallidus* | -4.053 |
| 5 | gi\|335059563 | Ribulose-1,5-bisphosphate carboxylase/oxygenase large subunit | 34.09/7.83 | 34.19/4.74 | 245 | 3 | *Humbertia madagascariensis* | -3.850 |
| 14 | gi\|502137718 | Predicted: ribulose bisphosphate carboxylase/oxygenase activase 2, chloroplastic-like | 48.07/8.47 | 40.24/5.02 | 298 | 7 | *Cicer arietinum* | 7.049 |
| 15 | gi\|488888859 | Chloroplast rubisco activase 1 | 48.37/7.66 | 39.76/5.09 | 300 | 4 | *Sagittaria graminea* | 2.472 |
| 21 | gi\|488888859 | Chloroplast rubisco activase 1 | 48.37/7.66 | 40.27/5.26 | 204 | 5 | *Sagittaria graminea* | 1.432 |
| 22 | gi\|355488628 | Ribulose bisphosphate carboxylase large chain | 19.46/4.87 | 48.33/5.43 | 72 | 4 | *Medicago truncatula* | -4.455 |
| 25 | gi\|452119476 | Ribulose bisphosphate carboxylase large subunit, partial (chloroplast) | 47.76/0.00 | 13.67/5.68 | 81 | 8 | *Ulva reticulata x Ulva taeniata* | -3.093 |
| 27 | gi\|355513999 | Phosphoribulokinase | 46.00/6.68 | 35.60/5.34 | 118 | 11 | *Medicago truncatula* | -1.498 |
| 28 | gi\|488888860 | Chloroplast rubisco activase 2 | 36.40/6.33 | 34.77/5.37 | 178 | 3 | *Sagittaria graminea* | 2.933 |
| 35 | gi\|488888859 | Chloroplast rubisco activase 1 | 48.37/7.66 | 35.45/5.50 | 283 | 4 | *Sagittaria graminea* | 2.256 |
| 36 | gi\|488888860 | Chloroplast rubisco activase 2 | 36.40/6.33 | 35.35/5.59 | 212 | 9 | *Sagittaria graminea* | 1.143 |
| 40 | gi\|340511990 | Ribulose-1,5-bisphosphate carboxylase/oxygenase large subunit, partial(chloroplast) | 50.77/6.23 | 45.85/5.62 | 337 | 14 | *Scutellaria minor* | -1.418 |
| 51 | gi\|413953335 | Transketolase isoform 2 | 69.06/5.46 | 68.55/6.07 | 167 | 4 | *Zea mays* | -1.502 |
| 66 | gi\|399139488 | Ribulose-1,5-bisphosphate carboxylase/oxygenase large subunit, partial | 51.66/6.19 | 45.56/6.70 | 435 | 14 | *Crossandra infundibuliformis* | 1.249 |
| Carbon metabolism | | |  |  |  |  |  |  |
| 13 | gi\|404551307 | Glyceraldehyde-3-phosphate dehydrogenase, partial | 13.46/6.89 | 49.62/6.82 | 133 | 1 | *Agave x ajoensis* | -2.462 |
| 47 | gi\|317373797 | Chloroplast phosphoglycerate kinase 3 | 50.28/6.69 | 46.51/5.79 | 284 | 8 | *Helianthus annuus* | -1.571 |
| 57 | gi\|449450436 | Predicted: glutamate-glyoxylate aminotransferase 2-like | 52.90/5.62 | 56.93/6.31 | 260 | 8 | *Cucumis sativus* | -3.996 |
| 61 | gi\|530684266 | Fructose-bisphosphate aldolase | 42.21/6.38 | 29.80/6.47 | 345 | 8 | *Oryza sativa* Japonica Group | 1.987 |
| 65 | gi\|332645863 | Triosephosphate isomerase | 27.38/5.39 | 19.18/4.00 | 151 | 4 | *Arabidopsis thaliana* | 4.485 |
| TCA cycle | |  |  |  |  |  |  |  |
| 45 | gi\|461488119 | Succinyl-CoA ligase beta-chain | 45.40/5.98 | 36.07/6.05 | 171 | 3 | *Oryza sativa* Japonica Group | 3.395 |
| 54 | gi\|433335660 | Malate dehydrogenase | 36.04/6.11 | 26.68/6.35 | 294 | 4 | *Brassica oleracea* | 1.040 |
| 62 | gi\|475577109 | Malate dehydrogenase 1, mitochondrial | 34.93/5.26 | 31.95/6.64 | 96 | 2 | *Aegilops tauschii* | 2.970 |
| Energy metabolism | |  |  |  |  |  |  |  |
| 16 | gi\|408899417 | AtpA, partial (chloroplast) | 55.13/5.53 | 50.63/5.10 | 315 | 12 | *Mammea americana* | 1.179 |
| 23 | gi\|408899391 | AtpA, partial (chloroplast) | 55.08/5.08 | 50.50/5.21 | 514 | 17 | *Erythroxylum areolatum* | -1.452 |
| 29 | gi\|546138044 | ATP synthase CF1 alpha subunit (chloroplast) | 56.14/5.70 | 47.94/5.38 | 163 | 5 | *Cocos nucifera* | -1.203 |
| 30 | gi\|402243685 | ATP synthase beta subunit, partial (chloroplast) | 49.69/5.11 | 61.11/5.41 | 737 | 9 | *Flindersia laevicarpa* | -3.030 |
| 31 | gi\|410176162 | ATP synthase CF1 beta subunit (chloroplast) | 53.54/5.09 | 55.39/5.44 | 939 | 19 | *Origanum vulgare* subsp. *vulgare* | -1.359 |
| 37 | gi\|223535342 | Alcohol dehydrogenase, putative | 41.61/8.61 | 32.30/5.63 | 103 | 3 | *Ricinus communis* | 4.043 |
| 53 | gi\|330254337 | NAD(P)-binding Rossmann-fold-containing protein | 34.97/8.37 | 21.16/6.41 | 102 | 3 | *Arabidopsis thaliana* | 1.887 |
| Amino acid and protein metabolism | | |  |  |  |  |  |  |
| 2 | gi\|226500014 | 3-beta hydroxysteroid dehydrogenase/isomerase family protein | 32.73/8.34 | 24.66/4.84 | 77 | 1 | *Zea mays* | -1.236 |
| 10 | gi\|222863465 | Glutamine synthetase family protein | 48.20/6.48 | 30.89/5.24 | 211 | 6 | *Populus trichocarpa* | 1.857 |
| 11 | gi\|223551115 | Proteasome subunit alpha type, putative | 30.64/4.89 | 32.86/5.24 | 192 | 8 | *Ricinus communis* | -2.136 |
| 17 | gi\|226499860 | Stromal 70 kDa heat shock-related protein | 74.85/5.08 | 73.19/4.96 | 259 | 6 | *Zea mays* | -2.046 |
| 32 | gi\|392465167 | Heat shock protein 70 | 71.46/5.14 | 66.46/5.31 | 140 | 4 | *Nicotiana tabacum* | 2.392 |
| 34 | gi\|527197598 | Cysteine synthase | 34.65/5.41 | 27.95/5.71 | 100 | 3 | *Genlisea aurea* | -3.840 |
| 38 | gi\|226508704 | Elongation factor Tu | 50.79/6.07 | 41.71/5.78 | 184 | 6 | *Zea mays* | -1.312 |
| 39 | gi\|449440632 | Predicted: elongation factor Tu, chloroplastic-like | 51.89/5.90 | 40.25/5.94 | 94 | 8 | *Cucumis sativus* | -1.458 |
| 41 | gi\|508784980 | TCP-1/cpn60 chaperonin family protein | 64.51/5.62 | 61.78/5.48 | 233 | 3 | *Theobroma cacao* | -1.794 |
| 44 | gi\|449506050 | Predicted: glutamine synthetase nodule isozyme-like | 39.30/5.59 | 36.26/6.00 | 75 | 4 | *Cucumis sativus* | 3.714 |
| 48 | gi\|543176708 | ATP sulfurylase 2-like protein | 40.12/0.00 | 45.36/5.86 | 253 | 11 | *Phaseolus vulgaris* | -1.428 |
| 50 | gi\|460415276 | Predicted: adenosyl homocysteinase-like | 53.69/5.78 | 51.09/6.03 | 355 | 9 | *Solanum lycopersicum* | -1.435 |
| 56 | gi\|350538867 | Arginase 2 | 36.94/5.60 | 36.83/6.37 | 193 | 8 | *Solanum lycopersicum* | 1.206 |
| Stress and defense | | |  |  |  |  |  |  |
| 1 | gi\|502111694 | Predicted: peroxiredoxin-2E, chloroplastic-like | 23.44/7.67 | 29.05/7.0 | 125 | 1 | *Cicer arietinum* | -1.189 |
| 4 | gi\|502111694 | Predicted: peroxiredoxin-2E, chloroplastic-like | 23.44/7.67 | 36.6/4.86 | 125 | 1 | *Cicer arietinum* | -5.574 |
| 7 | gi\|511774224 | 2-Cys peroxiredoxin, partial | 25.60/8.51 | 20.94/4.93 | 398 | 11 | *Nicotiana benthamiana* | -2.897 |
| 12 | gi\|223533515 | Peroxidase 12 precursor, putative | 39.40/7.55 | 35.09/4.94 | 110 | 2 | *Ricinus communis* | 1.136 |
| 24 | gi\|145323784 | L-ascorbate peroxidase 1 | 27.79/5.85 | 12.32/5.50 | 376 | 6 | *Arabidopsis thaliana* | -1.521 |
| 42 | gi\|460384911 | Predicted: annexin D5-like | 35.82/9.05 | 9.32/6.12 | 79 | 14 | *Solanum lycopersicum* | 2.169 |
| 46 | gi\|222856181 | Oxidoreductase family protein | 39.33/5.83 | 39.53/6.28 | 81 | 2 | *Populus trichocarpa* | 3.093 |
| 49 | gi\|508777590 | Monodehydroascorbate reductase 6 isoform 4 | 53.16/8.80 | 46.57/6.16 | 337 | 5 | *Theobroma cacao* | -1.598 |
| 60 | gi\|330255786 | Glutathione S-transferase phi 8 | 29.27/0.00 | 18.84/6.43 | 83 | 3 | *Arabidopsis thaliana* | 2.957 |
| Transcription and signal transduction | | |  |  |  |  |  |  |
| 8 | gi\|514824684 | Predicted: fanconi anemia group I protein homolog | 153.86/8.00 | 22.31/5.07 | 61 | 13 | *Setaria italica* | -1.455 |
| 9 | gi\|12229593 | RecName: Full=14-3-3-like protein | 29.35/4.79 | 21.17/5.03 | 96 | 2 | *Lilium longiflorum* | -1.349 |
| 18 | gi\|412993712 | Pre-mRNA-splicing factor 38A | 32.52/5.03 | 11.61/5.28 | 60 | 10 | *Bathycoccus prasinos* | -4.501 |
| 19 | gi\|474386333 | Poly(C)-binding protein 3 | 37.52/6.76 | 18.39/5.23 | 60 | 10 | *Triticum urartu* | 3.395 |
| 55 | gi\|449532425 | Predicted: chloroplast stem-loop binding protein of 41 kDa, chloroplastic-like, partial | 30.27/5.26 | 29.54/6.23 | 85 | 2 | *Cucumis sativus* | -1.761 |
| 58 | gi\|384582593 | Maturase K, partial (chloroplast) | 18.02/9.64 | 9.50/6.51 | 66 | 5 | *Poincianella exostemma* | -3.933 |
| 67 | gi\|527203530 | Actin-97 | 41.95/5.37 | 57.78/6.80 | 437 | 12 | *Genlisea aurea* | -1.757 |
| Unknown proteins | |  |  |  |  |  |  |  |
| 6 | gi\|388503040 | Unknown | 28.24/5.47 | 39.35/4.86 | 75 | 3 | *Medicago truncatula* | -3.176 |
| 59 | gi\|502142675 | Predicted: uncharacterized protein LOC101509967 Isoform X1 | 10.31/4.92 | 11.97/6.50 | 62 | 5 | *Cicer arietinum* | 3.827 |

^a^ The spot number corresponding to the number listed in the supplementary table 4. Underlined numbers represent *A. mollis* tissues.

^b^ Database accession numbers (gb) according to NCBInr.

^c^ The name of proteins was identiﬁed by LC-MALDI-TOF/TOF.

^d^ Theoretical mass (kDa) and p*I* of identiﬁed proteins. Theoretical values were retrieved from the NCBInr database.

^e^ Experimental mass (kDa) and p*I* of identiﬁed proteins. Experimental values were calculated by using PDquest software and standard molecular mass markers.

^f^ The Mascot searched score against the database NCBInr.

^g^ Number of matched peptide fragments.

^h^ The species that has the high homology of the identiﬁed protein.

^i^ Log_2_- (fold change) values between the different treatments. SF vs CK means soil flooding treatment vs control group.

**Supplementary Table 5.** Identiﬁcation of DEPs of *A. mollis* roots with an expression change greater than 2.0-fold change under tidal flooding stress

| Spot ^a^ | Accession (gb)^b^ | Protein Name ^c^ | Thero. ^d^ kDa/p*I* | Exper. ^e^ kDa/p*I* | Score^f^ | MP ^g^ | Species ^h^ | SF vs. CK ^i^ |
| --- | --- | --- | --- | --- | --- | --- | --- | --- |
| TCA cycle | | |  |  |  |  |  |  |
| R22 | gi\|113622845 | Os08g0120000 | 31.84/8.81 | 46.91/5.23 | 62 | 5 | *Oryza sativa* Japonica Group | 1.372 |
| R28 | gi\|470107271 | Predicted: succinyl-CoA ligase [ADP-forming] subunit beta, mitochondrial-like | 45.39/5.87 | 34.20/5.76 | 93 | 4 | *Fragaria vesca* subsp. *vesca* | -3.345 |
| R37 | gi\|475610756 | Succinate dehydrogenase (ubiquinone) flavoprotein subunit, mitochondrial | 81.40/6.24 | 57.35/6.09 | 98 | 5 | *Aegilops tauschii* | -2.810 |
| Carbon and energy metabolism | | |  |  |  |  |  |  |
| R3 | gi\|223547542 | NADH dehydrogenase, putative | 19.14/4.76 | 12.75/4.50 | 77 | 1 | *Ricinus communis* | -2.186 |
| R7 | gi\|223539983 | Alpha-galactosidase/alpha-n-acetylgalactosaminidase, putative | 40.06/5.19 | 40.56/4.28 | 125 | 6 | *Ricinus communis* | -1.844 |
| R10 | gi\|514802088 | Predicted: beta-glucosidase 12-like | 51.94/6.73 | 53.37/4.52 | 61 | 12 | *Setaria italica* | -3.641 |
| R20 | gi\|355479515 | Adenosine kinase | 38.08/5.08 | 25.56/5.20 | 103 | 6 | *Medicago truncatula* | 1.701 |
| R26 | gi\|470124373 | Predicted: beta-galactosidase 13-like | 87.36/8.91 | 60.62/5.27 | 63 | 9 | *Fragaria vesca* subsp. *vesca* | -6.185 |
| R30 | gi\|350538295 | Enolase | 48.05/5.68 | 49.90/5.73 | 301 | 8 | *Solanum lycopersicum* | 5.906 |
| R35 | gi\|550346968 | UDP-glucose 4-epimerase family protein | 38.44/5.66 | 31.38/5.99 | 108 | 7 | *Populus trichocarpa* | -2.385 |
| R36 | gi\|223525768 | Alcohol dehydrogenase, putative | 41.88/5.98 | 43.45/6.38 | 106 | 4 | *Ricinus communis* | 1.372 |
| R39 | gi\|390098824 | Triosephosphate isomerase cytosolic isoform-like | 27.31/5.72 | 16.51/4.00 | 207 | 6 | *Capsicum annuum* | -2.761 |
| R40 | gi\|11467928 | Cytochrome b | 45.52/9.85 | 24.80/6.69 | 61 | 3 | *Acutodesmus obliquus* | -2.804 |
| R42 | gi\|460405093 | Predicted: probable aldo-keto reductase 4-like | 60.97/5.51 | 31.89/6.22 | 76 | 5 | *Solanum lycopersicum* | -1.893 |
| R43 | gi\|508708397 | Thiamin diphosphate-binding fold (THDP-binding) superfamily protein isoform 2 | 36.04/8.67 | 35.88/6.31 | 63 | 5 | *Theobroma cacao* | -2.545 |
| R44 | gi\|223525768 | Alcohol dehydrogenase, putative | 41.88/5.98 | 43.26/6.32 | 61 | 5 | *Ricinus communis* | 2.574 |
| R45 | gi\|407369264 | Alcohol dehydrogenase, partial | 20.74/6.08 | 50.46/6.32 | 76 | 1 | *Pinus taiwanensis* | 1.412 |
| Amino acid and protein metabolism | | |  |  |  |  |  |  |
| R1 | gi\|460411270 | Predicted: 60S acidic ribosomal protein P2B-like isofoR1 | 11.41/4.55 | 9.28/4.05 | 86 | 1 | *Solanum lycopersicum* | -1.880 |
| R11 | gi\|432140649 | Heat shock protein 70 | 74.29/5.26 | 72.91/4.58 | 475 | 16 | *Lactuca sativa* | 3.076 |
| R13 | gi\|430763366 | Polyubiquitin 14, partial | 15.57/5.74 | 28.74/4.83 | 68 | 2 | *Cornus kousa* | -3.358 |
| R14 | gi\|502126081 | Predicted: proteasome subunit alpha type-1-B-like | 31.59/4.94 | 30.00/4.90 | 100 | 5 | *Cicer arietinum* | -1.714 |
| R15 | gi\|514761174 | Predicted: 26S protease regulatory subunit 6A homolog | 47.99/4.94 | 43.41/4.77 | 308 | 14 | *Setaria italica* | 1.053 |
| R23 | gi\|334184654 | Heat shock protein 60-2 | 61.78/6.08 | 60.46/5.14 | 202 | 11 | *Arabidopsis thaliana* | -3.003 |
| R24 | gi\|413956514 | Glutamine synthetase3 | 18.23/6.92 | 35.05/5.36 | 79 | 6 | *Zea mays* | -2.010 |
| R25 | gi\|330252829 | 20S proteasome alpha subunit G1 | 27.64/5.93 | 38.92/5.39 | 62 | 10 | *Arabidopsis thaliana* | 1.143 |
| R27 | gi\|508778822 | Tetratricopeptide repeat (TPR)-like superfamily protein, putative | 64.56/8.70 | 50.52/5.61 | 61 | 14 | *Theobroma cacao* | 1.036 |
| R29 | gi\|355429958 | Putative S-adenosyl-L-homocysteinase | 61.87/5.69 | 45.20/5.89 | 75 | 8 | *Linum usitatissimum* | 2.505 |
| R33 | gi\|527194033 | S-formylglutathione hydrolase, partial | 24.93/6.22 | 27.19/6.21 | 69 | 2 | *Genlisea aurea* | -2.060 |
| R38 | gi\|502137510 | Predicted: 60S ribosomal export protein NMD3-like | 59.49/6.07 | 15.16/6.32 | 71 | 14 | *Cicer arietinum* | -2.219 |
| Stress and defense | | |  |  |  |  |  |  |
| R2 | gi\|511774224 | 2-Cys peroxiredoxin, partial | 25.60/8.51 | 15.21/4.49 | 77 | 5 | *Nicotiana benthamiana* | -2.780 |
| R17 | gi\|440573478 | Tau class glutathione S-transferase | 27.80/7.62 | 48.05/4.92 | 63 | 8 | *Pinus tabuliformis* | -3.345 |
| Transcription and signal transduction | | |  |  |  |  |  |  |
| R4 | gi\|297333574 | GF14 omega | 29.37/4.70 | 21.33/4.35 | 112 | 4 | *Arabidopsis lyrata* subsp. *lyrata* | -2.804 |
| R8 | gi\|460377572 | Predicted: 14-3-3-like protein-like | 29.22/4.69 | 21.08/4.54 | 84 | 6 | *Solanum lycopersicum* | -1.246 |
| R9 | gi\|226498758 | Inositol-tetrakisphosphate 1-kinase 3 | 37.72/8.59 | 41.17/4.47 | 63 | 8 | *Zea mays* | -2.060 |
| R12 | gi\|413942896 | Profilin-4 | 14.21/4.63 | 10.66/4.71 | 71 | 2 | *Zea mays* | 4.448 |
| R21 | gi\|508715249 | Ran-binding protein 1 b isoform 1 | 25.21/4.70 | 29.56/4.99 | 79 | 2 | *Theobroma cacao* | -1.913 |
| R31 | gi\|508786508 | Cell division control 6 isoform 7 | 47.05/8.95 | 21.75/6.48 | 66 | 14 | *Theobroma cacao* | -1.658 |
| R32 | gi\|223549247 | ATP-dependent RNA helicase, putative | 78.24/8.86 | 23.64/6.31 | 62 | 16 | *Ricinus communis* | 1.741 |
| R34 | gi\|186510546 | Ankyrin repeat family protein | 87.56/6.52 | 26.05/6.27 | 67 | 11 | *Arabidopsis thaliana* | 2.089 |
| Photosynthesis | |  |  |  |  |  |  |  |
| R18 | gi\|543177187 | RuBisCO large subunit-binding protein subunit alpha, belongs to the chaperonin family | 61.28/0.00 | 54/4.8 | 147 | 4 | *Phaseolus vulgaris* | -1.076 |
| Other proteins | | |  |  |  |  |  |  |
| R6 | gi\|514802368 | Predicted: endochitinase A-like | 30.18/8.55 | 39.52/7.00 | 83 | 1 | *Setaria italica* | 3.202 |
| R16 | gi\|15242097 | Putative villin | 108.44/5.25 | 49.09/4.86 | 60 | 17 | *Arabidopsis thaliana* | -2.169 |
| R19 | gi\|508787331 | Ferritin 4 | 30.44/6.56 | 17.58/4.99 | 116 | 8 | *Theobroma cacao* | 5.694 |
| Unknown proteins | |  |  |  |  |  |  |  |
| R5 | gi\|300256770 | Hypothetical protein VOLCADRAFT_119802 | 87.21/5.31 | 32.42/4.25 | 61 | 9 | *Volvox carteri f. nagariensis* | -3.003 |
| R41 | gi\|162688983 | Predicted protein | 38.61/8.27 | 30.98/6.42 | 63 | 8 | *Physcomitrella patens* subsp. *patens* | -1.472 |

^a^ The spot number corresponding to the number listed in the supplementary table 5. R represents the root tissue*.* Underlined numbers represent *A. mollis* tissues.

^b^ Database accession numbers (gb) according to NCBInr.

^c^ The name of proteins was identiﬁed by LC-MALDI-TOF/TOF.

^d^ Theoretical mass (kDa) and p*I* of identiﬁed proteins. Theoretical values were retrieved from the NCBInr database.

^e^ Experimental mass (kDa) and p*I* of identiﬁed proteins. Experimental values were calculated by using PDquest software and standard molecular mass markers.

^f^ The Mascot searched score against the database NCBInr.

^g^ Number of matched peptide fragments.

^h^ The species that has the high homology of the identiﬁed protein.

^i^ Log_2_- (fold change) values between the different treatments. SF vs CK means soil flooding treatment vs control group.

**Supplementary Table 7.** The percentage of functional classification analysis of DEPs of *A. ilicifolius* and *A. mollis*

| **Functional classification** | ***A.ilicifolius*** | | ***A.mollis*** | |
| --- | --- | --- | --- | --- |
|  | Leaf | Root | Leaf | Root |
| Photosynthesis and photorespiration | 15.385% | 0% | 14.925% | 0% |
| TCA cycle | 3.846% | 2.5% | 4.478% | 2.222% |
| Carbon and energy metabolism | 21.795% | 25% | 7.463% | 11.111% |
| Amino acid and protein metabolism | 15.385% | 20% | 5.970% | 11.111% |
| Stress and defense | 1.282% | 10% | 5.970% | 0% |
| Transcription and signal transduction | 11.538% | 12.5% | 1.493% | 6.667% |
| Other proteins | 2.564% | 2.5% | 0% | 4.444% |
| Unknown proteins | 0% | 5% | 1.493% | 0% |
| Photosynthesis and photorespiration | 8.974% | 0% | 16.418% | 2.222% |
| TCA cycle | 0% | 0% | 0% | 4.444% |
| Carbon and energy metabolism | 3.846% | 2.5% | 10.448% | 20% |
| Amino acid and protein metabolism | 11.538% | 2.5% | 13.433% | 15.556% |
| Stress and defense | 1.282% | 2.5% | 7.463% | 4.444% |
| Transcription and signal transduction | 2.564% | 5% | 8.955% | 11.111% |
| Other proteins | 0% | 2.5% | 0% | 2.222% |
| Unknown proteins | 0% | 1.493% | 7.5% | 4.444% |

Red represents up-regulated; blue represents down-regulated.
